# Application of targeted panel sequencing and whole exome sequencing for 76 Chinese families with retinitis pigmentosa

**DOI:** 10.1002/mgg3.1131

**Published:** 2020-01-20

**Authors:** Handong Dan, Xin Huang, Yiqiao Xing, Yin Shen

**Affiliations:** ^1^ Eye Center Renmin Hospital of Wuhan University Wuhan Hubei China

**Keywords:** gene variant, next‐generation sequencing, retinitis pigmentosa, targeted panels sequencing, whole exome sequencing

## Abstract

**Background:**

This study aimed to identify the gene variants and molecular etiologies in 76 unrelated Chinese families with retinitis pigmentosa (RP).

**Methods:**

In total, 76 families with syndromic or nonsyndromic RP, diagnosed on the basis of clinical manifestations, were recruited for this study. Genomic DNA samples from probands were analyzed by targeted panels or whole exome sequencing. Bioinformatics analysis, Sanger sequencing, and available family member segregation were used to validate sequencing data and confirm the identities of disease‐causing genes.

**Results:**

The participants enrolled in the study included 62 families that exhibited nonsyndromic RP, 13 that exhibited Usher syndrome, and one that exhibited Bardet–Biedl syndrome. We found that 43 families (56.6%) had disease‐causing variants in 15 genes, including *RHO, PRPF31, USH2A, CLRN1, BBS2, CYP4V2, EYS, RPE65, CNGA1, CNGB1, PDE6B, MERTK, RP1, RP2,* and *RPGR*; moreover, 12 families (15.8%) had only one heterozygous variant in seven autosomal recessive RP genes, including *USH2A*, *EYS*, *CLRN1*, *CERKL*, *RP1*, *CRB1*, and *SLC7A14*. We did not detect any variants in the remaining 21 families (27.6%). We also identified 67 potential pathogenic gene variants, of which 24 were novel.

**Conclusion:**

The gene variants identified in this study expand the variant frequency and spectrum of RP genes; moreover, the identification of these variants supplies foundational clues for future RP diagnosis and therapy.

## Graphical abstract

The gene variants identified in this study expand the variant frequency and spectrum of RP genes. The identification of these variants supplies foundational clues for future RP diagnosis and therapy.

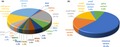

## 1 INTRODUCTION

Retinitis pigmentosa (RP; OMIM # 268000) is a clinically and genetically heterogeneous inherited retinal dystrophy (Huang, Wu, Lv, Zhang, & Jin, 2015; Lee & Garg, 2015). It is characterized by the progressive loss of rod and cone photoreceptors, which leads to severe visual dysfunction in bilateral eyes (Hartong, Berson, & Dryja, 2006). Typical symptoms include progressive night blindness, loss of vision, and tunnel vision. The prevalence of RP is approximately one in 750–9000 individuals (Na et al., 2017); RP affects approximately 2.5 million people worldwide (Dias et al., 2018). Affected individuals can inherit RP in one of the following patterns: autosomal dominant (adRP, 15%–25%), autosomal recessive (arRP, 5%–20%), X‐linked (xlRP, 5%–15%), or unknown (40%–50%) (Ferrari et al., 2011; Lipinski, Thake, & MacLaren, 2013; Oishi et al., 2014). RP is categorized as either of two types: nonsyndromic or syndromic. Approximately 20%–30% of patients are presumed to exhibit syndromic RP (Dias et al., 2018). Variants in genes that are primarily expressed in retinal cells result in nonsyndromic RP; conversely, variants in genes expressed in a variety of cells or tissues lead to syndromic RP (Waters & Beales, 2011; Wheway, Parry, & Johnson, 2014), such as Usher syndrome or Bardet–Biedl syndrome.

Thus far, 98 genes (33 for syndromic RP and 65 for nonsyndromic RP) and 9 loci (3 for syndromic RP and 6 for nonsyndromic RP) are known to cause RP. More than 3,000 gene variants are responsible for nonsyndromic RP (Guadagni, Novelli, Piano, Gargini, & Strettoi, 2015). The underlying molecular etiologies involve the phototransduction cascade and retinal transcription factors associated with the phototransduction cascade, as well as ribonucleic acid splicing machinery, retinal metabolism, retinal cell structure, ciliary structure, and ciliary function (Veleri et al., 2015). Most genes associated with RP are expressed in rod photoreceptors, whereas a small number are expressed in retinal pigment epithelium (Koch et al., 2012). Next‐generation sequencing (NGS) technology in bioinformatics and computing technologies has undergone rapid development; accordingly, low‐cost, high‐throughput, highly efficient DNA sequencing has enabled accurate diagnosis and precise assessment of patient prognosis. Inherited genetic diseases are increasingly diagnosed accurately using NGS technology (Bamshad et al., 2011; Bell et al., 2011; Neuhaus et al., 2017; Yang et al., 2013). However, it remains a considerable challenge to identify disease‐causing genes with NGS technology (Bainbridge et al., 2008). Inherited gene variants are reportedly responsible for only 60% of known cases of RP (Huang et al., 2017; Xu et al., 2014; Zhang, 2016); thus, the disease‐causing gene is unknown in a substantial proportion of affected individuals. It is imperative to determine the genetic etiology of RP and provide guidance for efficient molecular diagnosis.

In this study, we enrolled 76 families with syndromic or nonsyndromic RP. All probands were evaluated using NGS technology. Through functional prediction, Sanger sequencing, and segregation analysis, we found that 43 families (56.6%) had disease‐causing variants in 15 genes, while 12 families (15.8%) had only 1 heterozygous variant in 7 arRP genes. We also identified 67 potential pathogenic gene variants, of which 24 have not been previously described.

## 2 MATERIALS AND METHODS

### 2.1 Ethical compliance

The research protocol was approved by the medical ethics committee of Renmin Hospital of Wuhan University and carried out in accordance with the tenets of the Declaration of Helsinki. Written informed consent was obtained from each participant or their guardian (for participants who were children) prior to the study. All participants were consecutively recruited in Renmin Hospital of Wuhan University (Hubei, China), which is located in central China.

### 2.2 Clinical testing

A detailed family history was obtained from the proband or the proband's family members. All participants received comprehensive ophthalmological examinations, including best‐corrected visual acuity, refractive error measurement, slit lamp examination, intraocular pressure measurement, and funduscopy. Participants who agreed to additional ophthalmological examinations underwent fundus photography, visual field assessment, optical coherence tomography (OCT), and full‐field electroretinography (ERG). High‐resolution fundus photographs were obtained with a digital fundus camera VISUCAM 200 (Carl Zeiss Meditec AG, Jena, Thuringia, Germany). Visual field assessment was performed using a Humphrey HFA II‐750 (Carl Zeiss Meditec AG). OCT was performed using an AngioVue® Imaging System (Optovue). ERG was recorded using an Espion system (Diagnosys) in accordance with the standards and methodology of the International Society for Clinical Electrophysiology of Vision (Mcculloch et al., 2015). Participants who exhibited hearing loss or carried gene variants indicative of Usher syndrome underwent hearing examinations using an ITERA sonometer (Otometrics, DK‐2630).

### 2.3 Targeted panel sequencing and whole exome sequencing

Genomic DNA was analyzed with targeted panel sequencing (each of six panels containing 70, 316, 78, 370, 429, and 386 genes) or whole exome sequencing (WES). Genes included in the panels are listed in Text S1; these genes are primarily responsible for inherited retinal dystrophy. Genomic DNA was isolated from leukocytes of venous blood samples using the QIAamp DNA Blood Midi Kit (Qiagen) or TIANamp Blood DNA Midi Kit (TIANGEN Biotech), in accordance with the manufacturer's standard protocol. Library preparation was performed using the Ion AmpliseqTM Library Kit 2 or SureSelect Exome V5 Capture library, in accordance with the manufacturer's instructions (Biswas et al., 2017; Chen et al., 2013; Javadiyan et al., 2018). Sequencing was performed on an Ion Torrent PGM (Life Technologies) or HiSeq (Illumina) platform.

### 2.4 Data analysis

The variant nomenclature used in this study complied with the recommendations of the Human Genomic Variation Society (HGVS, http://www.hgvs.org/) (Wang et al., 2018). Sequence alignments were performed using the Torrent Suite or Burrows‐Wheeler Aligner (Li & Durbin, 2010). Variant calling and annotation were conducted in accordance with a previously published protocol (Liu et al., 2015; Siggs et al., 2017). The raw reads were filtered as clean reads and then aligned to the GRCh37 (hg19) human reference sequence. Variants were preferentially selected for further analysis and validation if they met the following criteria: (a) their minor allele frequency <0.01 in the 1,000 Genomes Project database (http://www.internationalgenome.org/), Exome Aggregation Consortium database (ExAC, http://exac.broadinstitute.org/), Genome Aggregation database (gnomAD, http://gnomad.broadinstitute.org/), Single Nucleotide Polymorphisms database (dbSNP, https://www.ncbi.nlm.nih.gov/snp), and in‐house database with exomes of Chinese individuals; (b) they were nonsynonymous; (c) they were located in exon or intron regions that affected RNA splicing; (d) they were predicted to be damaging or deleterious variants using Polymorphism Phenotyping (PolyPhen2, http://genetics.bwh.harvard.edu/pph2/) (Adzhubei et al., 2010) and Sorting Intolerant From Tolerant (SIFT, http://sift.jcvi.org/) (Kumar, Henikoff, & Ng, 2009). Variant annotation in this study complied with the guidelines of the American College of Medical Genetics (ACMG, https://www.acmg.net/) (ACMG Board of Directors, 2016; Richards et al., 2015). Conservation of each amino acid substitution was calculated using PhyloP in Mutation Taster (http://www.mutationtaster.org/) (Schwarz, Cooper, Schuelke, & Seelow, 2014). A PhyloP value between −14 and +6 was considered indicative of amino acid is conservation among different species. Molecular modeling of wild‐type and mutant protein sequences were computed by a SWISS‐MODEL server homology modeling pipeline that relies on ProMod3, an in‐house comparative modeling engine based on OpenStructure (Bertoni, Kiefer, Biasini, Bordoli, & Schwede, 2017; Bienert et al., 2017; Waterhouse et al., 2018).

### 2.5 Sanger sequencing and segregation analysis

Raw reads were filtered and the selected variants were subjected to validation and segregation analyses. Polymerase chain reaction was used to amplify gene fragments that included the variants. Primers were designed with Primer3 (http://primer3.ut.ee/); primers used for Sanger sequencing are listed in Table S2. The amplicons were sequenced using 3500xL Dx Genetic Analyser (Applied Biosystems, Foster City, CA, USA) with ABI BigDye Terminator v3.1 Cycle Sequencing kit. The proband sequences and corresponding consensus sequences (obtained from the NCBI Human Genome Database https://www.ncbi.nlm.nih.gov/) were analyzed using the SeqMan II software of the Lasergene software package (DNASTAR). DNA samples of all probands and their available family members were subjected to Sanger sequencing and segregation analysis based on the inheritance pattern.

## 3 RESULTS

### 3.1 Clinical manifestations

In total, 76 Chinese families of Han ethnicity were consecutively enrolled in the study. All probands complained of night blindness, constricted vision field, and impaired vision, with the exception of proband 12, who was very young. Four probands who exhibited RP beginning in childhood had complained of strabismus and nystagmus. Most probands exhibited fundus signs typical of RP, including bone spicule pigmentation, retinal vascular stenosis, and waxy‐pale optic disc. The fundus photographs of probands with novel variants are shown in Figure S1. Visual field analyses showed that probands had a constricted visual field with increased mean deviation. OCT revealed severe thinning of the retinal nerve fiber layer, outer nuclear layer, and epiretinal membranes. Full‐field ERG demonstrated extinguished or severely reduced dark‐adapted and light‐adapted responses, with significant reductions of a and b waves. Typical visual field, OCT, and ERG are shown in Figure S2. Clinical features of the 43 probands with disease‐causing genes are listed in Table 1.

**Table 1:** Clinical features of probands with disease‐causing genes

No.ID	Gender	Inheritance	Segregation	Clinical manifestations	Age at (year)		BCVA		Fundus Examination	mRNFL (um)		Visual Field (mean deviation)		ERG	
					Onset	Exam	OD	OS		OD	OS	OD	OS	OD	OS
127	M	AD	Yes	NB, VFD, VD	12	42	FC	FC	BSPD, ARA, WOD	195	177	NA	NA	NA	NA
128	M	AD	NA	NB, VFD, VD	14	42	FC	HM	BSPD, ARA, WOD	199	188	NA	NA	NA	NA
133	F	AD	NA	NB, VFD, VD	6	36	0.2	0.08	BSPD, ARA, WOD	137	113	27.12	29.3	NA	NA
1	M	S	NA	NB, VFD, VD, SNHL	15	21	0.6	0.6	BSPD, ARA, WOD	NA	NA	25.47	25.87	NA	NA
3	M	S	NA	NB, VFD, VD	25	47	0.4	0.4	BSPD, ARA, WOD	150	156	26.84	27.49	E	E
17	M	S	NA	NB, VFD, VD, SNHL	40	59	0.1	0.12	BSPD, ARA, WOD	NA	NA	27.65	28.51	E	E
21	M	S	Yes	NB, VFD, VD, SNHL	14	34	0.6	0.8	BSPD, ARA, WOD	183	169	27.08	28.5	NA	NA
27	M	AR	Yes	NB, VFD, VD, SNHL	13	21	0.8	0.6	PD, ARA, WOD	223	211	28.22	27.88	E	E
37	F	S	NA	NB, VFD, VD	25	44	0.12	0.05	BSPD, ARA, WOD	113	132	16.65	18.08	NA	NA
49	F	S	Yes	NB, VFD, VD, SNHL	4	20	0.6	0.8	PD, ARA, WOD	321	350	24.26	25.5	E	E
67	M	S	NA	NB, VFD, VD, SNHL	20	31	0.1	0.08	PD, ARA, WOD, MD	168	196	30.54	30.64	E	E
109	M	S	NA	NB, VFD, VD, SNHL	16	46	0.4	0.2	BSPD, ARA, WOD	192	201	25.56	27.89	NA	NA
113	F	S	Yes	NB, VFD, VD	20	40	HM	HM	BSPD, ARA, WOD	189	185	NA	NA	NA	NA
117	M	S	NA	NB, VFD, VD, SNHL	33	43	0.12	0.05	BSPD, ARA, WOD	194	193	27.72	26.49	E	E
118	M	AR	NA	NB, VFD, VD, SNHL	30	50	0.1	0.25	BSPD, ARA, WOD	153	148	NA	NA	NA	NA
146	F	AR	NA	NB, VFD, VD, SNHL	22	44	LP	LP	BSPD, ARA, WOD	NA	NA	NA	NA	NA	NA
154	M	S	Yes	NB, VFD, VD	20	36	0.6	0.8	BSPD, ARA, WOD	209	213	27.8	28.38	E	E
173	M	AR	Yes	NB, VFD, VD	25	46	HM	HM	BSPD, ARA, WOD	NA	NA	NA	NA	E	E
164	M	AR	Yes	NB, VFD, VD, SNHL	5	40	0.1	0.12	BSPD, ARA, WOD	159	162	27.97	28.62	NA	NA
28	M	S	Yes	NB, VFD, VD	4	26	HM	HM	TLR, ARA, WOD	191	188	NA	NA	E	E
13	M	S	NA	NB, VFD, VD	14	54	LP	LP	SP, ARA, WOD	NA	NA	NA	NA	E	E
55	F	S	Yes	NB, VFD, VD	22	36	0.05	0.1	SP, ARA, WOD	215	239	NA	NA	NA	NA
74	F	S	NA	NB, VFD, VD	40	53	0.5	0.4	SP, PD, ARA, WOD	175	185	28.1	29.39	E	E
93	M	S	NA	NB, VFD, VD	25	37	0.3	0.15	SP, PD, ARA, WOD	194	181	NA	NA	NA	NA
132	M	AR	NA	NB, VFD, VD	25	56	HM	LP	SP, PD, ARA, WOD	234	153	NA	NA	NA	NA
7	M	S	NA	NB, VFD, VD	19	54	0.2	0.5	BSPD, ARA, WOD	172	172	29.59	29.14	E	E
62	F	S	NA	NB, VFD, VD	45	64	FC	0.12	BSPD, ARA, WOD	156	187	NA	NA	E	E
112	M	S	NA	NB, VFD, VD	30	36	0.1	0.12	BSPD, ARA	174	195	29.12	30	E	E
135	M	S	Yes	NB, VFD, VD	8	9	0.6	0.15	TLR	NA	NA	29.7	31.64	E	E
96	M	S	Yes	NB, VFD, VD, N, S	5	25	LP	HM	BSPD, ARA, WOD	NA	NA	NA	NA	NA	NA
143	M	S	Yes	NB, VFD, VD, N, S	5	31	LP	LP	BSPD, ARA, WOD, MD	NA	NA	NA	NA	NA	NA
165	M	S	Yes	NB, VFD, VD, N, S	6	28	LP	LP	BSPD, ARA, WOD	NA	NA	NA	NA	NA	NA
16	F	S	Yes	NB, VFD, VD	15	29	0.8	1	TLR	254	252	22.02	21.11	E	E
58	M	S	Yes	NB, VFD, VD	35	55	HM	HM	BSPD, ARA, WOD	NA	NA	NA	NA	E	E
64	F	S	Yes	NB, VFD, VD	35	46	0.1	0.1	BSPD, ARA, WOD	159	175	28.43	26.67	E	E
152	M	S	Yes	NB, VFD, VD	25	37	0.8	0.8	ARA, TLR	NA	NA	30.94	31.24	E	E
168	F	S	Yes	NB, VFD, VD	18	39	0.25	0.25	BSPD, ARA, WOD	168	179	27.56	26.45	E	E
157	F	AR	Yes	NB, VFD, VD, N, S	6	30	HM	HM	BSPD, ARA, WOD	NA	NA	NA	NA	NA	NA
12	M	XL	Yes	VD	4	7	0.5	0.5	TLR	NA	NA	NA	NA	NA	NA
79	M	S	Yes	NB, VFD, VD, N, S	10	39	LP	LP	BSPD, ARA, WOD	NA	NA	NA	NA	NA	NA
15	M	S	No	NB, VFD, VD	27	37	0.1	0.3	BSPD, ARA, WOD	148	146	30.15	30.2	E	E
68	M	S	NA	NB, VFD, VD	38	51	0.1	0.1	BSPD, ARA, WOD	143	154	NA	NA	NA	NA
176	M	S	No	NB, VFD, VD	8	29	0.1	0.3	BSPD, ARA, WOD	170	165	28.04	28.96	E	E

Abbreviations: AD, autosomal dominant; ARA, attenuated retinal arteries; AR, autosomal recessive; BCVA, best‐corrected visual acuity; BSPD, bone spicule pigmentation deposit; E, extinguished; ERG, electroretinography; F, female; FC, finger counting; HM, hand movement; LP, light perception; M, male; MD, macular degeneration; mRNFL, mean retinal nerve fiber layer; N, Nystagmus no; NA, not available; NB, night blindness; OD, right eye; OS, left eye; PD, pigmentation deposit; S, sporadic; S, Strabismus; SNHL, sensorineural hearing loss; SP, salt‐and‐pepper‐like retinal degeneration; TLR, tapetal‐like retinal degeneration; VD, vision decline; VFD, vision field defect; WOD, waxy‐pale optic disc; XL, X‐linked.

In total, 15 probands harbored *USH2A* (OMIM * 608400) compound heterozygous or homozygous variants, while 1 proband harbored *CLRN1* (OMIM * 606397) homozygous variants and 3 probands harbored *USH2A* heterozygous variants. Thirteen probands (11 probands with compound heterozygous or homozygous variants and two probands with *USH2A* heterozygous variants) were diagnosed with Usher syndrome. Six probands (five probands with *USH2A* compound heterozygous or homozygous variants and one proband with *USH2A* heterozygous variants) did not complain of hearing loss and did not exhibit hearing impairment in hearing examinations; they were diagnosed with nonsyndromic RP. Proband 28 had a compound heterozygous *BBS2* (OMIM * 606151) variant and was diagnosed with Bardet–Biedl syndrome; he exhibited fourth toe brachydactyly in both feet, which was more severe in the right foot. The proband exhibited obesity, with a body mass index of 28.2 kg/m^2^; he refused further examinations (e.g., sperm or genital gland). Notably, he did not exhibit obvious bone spicule pigmentation in the fundus and showed no mental retardation. Five probands with *CYP4V2* (OMIM * 608614) compound heterozygous or homozygous variants were diagnosed with Bietti crystalline corneoretinal dystrophy. They exhibited typical RP fundus performance with salt‐and‐pepper‐like retinal degeneration.

### 3.2 NGS results

**Figure 1:**
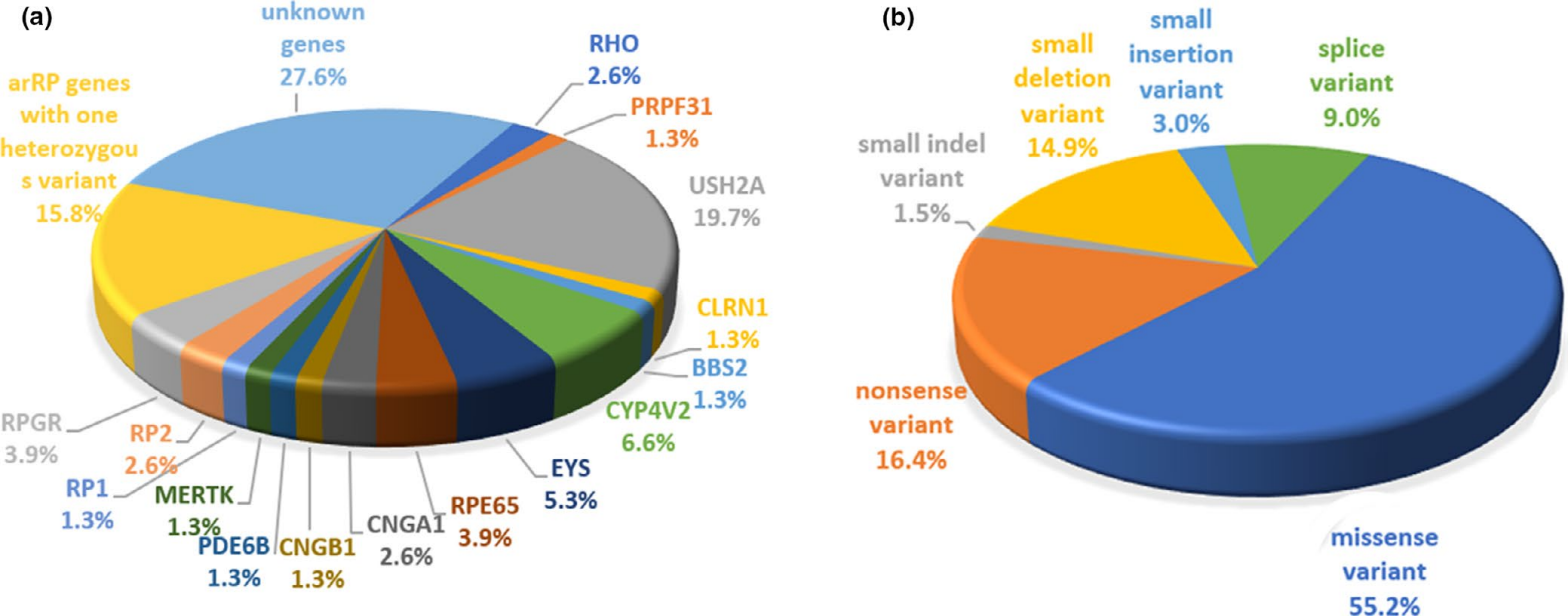
Spectrograms of genes and variants for RP probands. (a) Proportions of genes associated with retinitis pigmentosa (RP). (b) Proportions of all types of variants

Based on bioinformatics, Sanger sequencing validation, and segregation analysis, we found that 43 families (56.6%) had disease‐causing variants in 15 genes, including *RHO* (OMIM * 180380)*, PRPF31* (OMIM * 606419)*, USH2A, CLRN1, BBS2, CYP4V2, EYS* (OMIM * 612424)*, RPE65* (OMIM * 180069)*, CNGA1* (OMIM * 123825)*, CNGB1* (OMIM * 600724)*, PDE6B* (OMIM * 180072)*, MERTK* (OMIM * 604705)*, RP1* (OMIM * 603937)*, RP2* (OMIM * 300757)*,* and *RPGR* (OMIM * 312610). Segregation analysis was available for 24 of the 43 families, and the variants were segregated with the disease, except for Family 15 and Family 176. Two genes were associated with adRP in three families with heterozygous variants; 11 genes were associated with arRP in 35 families with homozygous variants (10 families) or compound heterozygous variants (25 families); and 2 genes were associated with xlRP in 5 families with hemizygous variants. The gene most frequently found in the study is *USH2A* (19.7%), followed by *CYP4V2* (6.6%). The gene variants of these probands are described in Table 2. The genomic information is shown in Table S3. In addition, we found that 12 families (15.8%) had only one heterozygous variant in seven arRP genes, including *USH2A, EYS, CLRN1, CERKL* (OMIM * 608381)*, RP1, CRB1* (OMIM * 604210)*,* and *SLC7A14* (OMIM * 615720); these heterozygous variants are described in Table 3. We did not detect any variants in the remaining 21 families (27.6%). The proportions of genes associated with RP in this cohort are shown in Figure 1a.

**Table 2:** Variant information of disease‐causing genes was detected in the study

**No. ID**	Disease	Panel	Gene	Nucleotide change	Amino acid change	Variant type	Exon/Intron	Hom/Het/Hem	Polyphen2	SIFT	PhyloP	Reference	ACMG
127	RP	Panel 2	RHO	c.1045T>C	p.(*349Glnnext*51)	nonsense	E5	Het	—	—	4.658	PMID:24705292	P
128	RP	WES	RHO	c.1040C>T	p.(Pro347Leu)	missense	E5	Het	PrD	D	5.624	PMID:22217031	P
133	RP	Panel 2	PRPF31	c.220C>T	p.(Gln74*)	nonsense	E3	Het	—	—	4.986	PMID:16799052	P
1	Usher	Panel 1	USH2A	c.538T>C	p.(Ser180Pro)	missense	E3	Het	PrD	D	3.592	PMID:19737284	LP
			USH2A	c.11714G>C	p.(Arg3905Pro)	missense	E61	Het	PrD	D	5.607	Novel	UVS
3	RP	Panel 3	USH2A	c.142_143insGA	p.(Lys48Argfs*98)	insertion	E2	Het	—	—	0.524	PMID:30076350	P
			USH2A	c.2802T>G	p.(Cys934Trp)	missense	E13	Het	PrD	D	0.999	PMID:25356976	LP
17	Usher	Panel 1	USH2A	c.11156G>A	p.(Arg3719His)	missense	E57	Hom	PrD	D	2.111	PMID:28157192	LP
21	Usher	Panel 3	USH2A	c.4165delG	p.(Val1389Leufs*43)	deletion	E19	Het	—	—	−0.137	PMID:30076350	LP
			USH2A	c.11156G>A	p.(Arg3719His)	missense	E57	Het	PrD	D	2.111	PMID:28157192	LP
27	Usher	Panel 1	USH2A	c.4645C>T	p.(Arg1549*)	nonsense	E22	Het	—	—	1.336	PMID:26352687	P
			USH2A	c.8559‐2A>G	—	splice	I42	Het	—	—	‐	PMID:25078356	P
37	RP	Panel 1	USH2A	c.1397G>T	p.(Gly466Val)	missense	E8	Hom	PrD	D	5.667	PMID:24938718	LP
49	Usher	Panel 2	USH2A	c.656A>C	p.(His219Pro)	missense	E4	Het	PoD	D	3.544	Novel	UVS
			USH2A	c.11208_11209insT	p.(Lys3737*)	insertion	E57	Het	—	—	1.194	Novel	LP
67	Usher	Panel 5	USH2A	c.2017T>A	p.(Cys673Ser)	missense	E12	Hom	PrD	D	4.591	Novel	UVS
109	Usher	WES	USH2A	c.8559‐2A>G	—	splice	I42	Het	—	—	‐	PMID:25078356	P
			USH2A	c.1143G>C	p.(Gln381His)	missense	E6	Het	PrD	N	6.022	Novel	UVS
113	RP	Panel 5	USH2A	c.2802T>G	p.(Cys934Trp)	missense	E13	Het	PrD	D	0.999	PMID:25356976	LP
			USH2A	c.4616C>T	p.(Thr1539Ile)	missense	E21	Het	PrD	N	4.998	PMID:30029497	UVS
117	Usher	Panel 5	USH2A	c.475C>T	p.(Gln159*)	nonsense	E2	Het	—	—	3.108	Novel	LP
			USH2A	c.8559‐2A>G	—	splice	I42	Het	—	—	‐	PMID:25078356	P
118	Usher	WES	USH2A	c.11156G>A	p.(Arg3719His)	missense	E57	Het	PrD	D	2.111	PMID:28157192	P
			USH2A	c.8559‐2A>G	—	splice	I42	Het	—	—	‐	PMID:25078356	P
146	Usher	Panel 6	USH2A	c.8559‐2A>G	—	splice	I42	Het	—	—	‐	PMID:25078356	P
			USH2A	c.14426C>T	p.(Thr4809Ile)	missense	E66	Het	PrD	D	6.161	PMID:18665195	LP
154	RP	Panel 6	USH2A	c.11156G>A	p.(Arg3719His)	missense	E57	Het	PrD	D	2.111	PMID:28157192	LP
			USH2A	c.9958G>T	p.(Gly3320Cys)	missense	E50	Het	PrD	D	5.589	PMID:25133613	LP
173	RP	Panel 6	USH2A	c.10588C>A	p.(Pro3530Thr)	missense	E54	Het	B	N	0.482	Novel	UVS
			USH2A	c.13339A>G	p.(Met4447Val)	missense	E63	Het	B	D	1.334	PMID:29625443	UVS
164	Usher	Panel 6	CLRN1	c.253+6T>C	—	splice	I1	Hom	—	—	‐	PMID:25356976	LP
28	RP	Panel 2	BBS2	c.563delT	p.(Ile188Thrfs*13)	deletion	E5	Het	—	—	3.233	PMID:24608809	P
			BBS2	c.1237C>T	p.(Arg413*)	nonsense	E11	Het	—	—	2.828	PMID:12920096	P
13	Bietti	Panel 3	CYP4V2	c.802‐6_810delATACAGGTCATCGCT	—	deletion	I6‐E7	Hom	—	—	‐	PMID:30076350	P
55	Bietti	Panel 2	CYP4V2	c.992A>C	p.(His331Pro)	missense	E8	Hom	PrD	D	4.751	PMID:22772592	P
74	Bietti	Panel 2	CYP4V2	c.802‐6_810delATACAGGTCATCGCT	—	deletion	I6‐E7	Het	—	—	‐	PMID:30076350	P
			CYP4V2	c.1199G>A	p.(Arg400His)	missense	E9	Het	PrD	D	−0.223	PMID:16179904	LP
93	Bietti	WES	CYP4V2	c.1091‐2A>G	—	splice	I8	Het	—	—	‐	PMID:25356976	P
			CYP4V2	c.802‐8_810delTCATACAGGTCATCGCG/insGC	—	indel	I6‐E7	Het	—	—	‐	PMID:23793346	P
132	Bietti	WES	CYP4V2	c.413G>A	p.(Ser138Asn)	missense	E3	Het	PrD	D	0.147	Novel	UVS
			CYP4V2	c.992A>C	p.(His331Pro)	missense	E8	Het	PrD	D	4.751	PMID:25356976	P
7	RP	Panel 3	EYS	c.8545C>T	p.(Arg2849*)	nonsense	E43	Het	—	—	2.49	PMID:30076350	P
			EYS	c.5644+5G>A	—	splice	I26	Het	—	—	‐	PMID:30076350	P
62	RP	Panel 1	EYS	c.2953_2961delACTGATGGA	p.(Thr985_Gly987del)	deletion	E19	Het	—	—	0.17	PMID:29159838	LP
			EYS	c.8805C>A	p.(Tyr2935*)	nonsense	E43	Het	—	—	0.382	PMID:28763560	P
112	RP	Panel 6	EYS	c.4955C>A	p.(Ser1652*)	nonsense	E26	Het	—	—	2.076	PMID:28559085	P
			EYS	c.6557G>A	p.(Gly2186Glu)	missense	E32	Het	PoD	D	0.561	PMID:25356976	LP
135	RP	Panel 2	EYS	c.9209T>C	p.(Ile3070Thr)	missense	E43	Het	B	N	1.839	PMID:26161267	LP
			EYS	c.3489T>A	p.(Asn1163Lys)	missense	E23	Het	PrD	D	1.174	PMID:22302105	LP
96	RP	Panel 1	RPE65	c.131G>A	p.(Arg44Gln)	missense	E3	Hom	PrD	D	5.775	PMID:25775262	LP
143	RP	WES	RPE65	c.725+2T>A	—	splice	I7	Hom	—	—	‐	Novel	LP
165	RP	Panel 6	RPE65	c.1379G>A	p.(Trp460*)	nonsense	E13	Het	—	—	5.985	Novel	LP
			RPE65	c.1403C>T	p.(Ser468Leu)	missense	E13	Het	PrD	D	5.985	Novel	UVS
16	RP	Panel 3	CNGA1	c.829G>A	p.(Asp277Asn)	missense	E9	Het	PrD	D	5.52	PMID:30652268	P
			CNGA1	c.472delC	p.(Leu158Phefs*4)	deletion	E5	Het	—	—	2.191	PMID:26496393	P
58	RP	Panel 4	CNGA1	c.472delC	p.(Leu158Phefs*4)	deletion	E5	Hom	—	—	2.191	PMID:26496393	P
64	RP	Panel 4	CNGB1	c.2921T>G	p.(Met974Arg)	missense	E29	Hom	PrD	D	3.182	Novel	UVS
152	RP	Panel 6	PDE6B	c.622G>A	p.(Val208Met)	missense	E3	Het	PoD	N	0.065	Novel	UVS
			PDE6B	c.2435A>T	p.(Asp812Val)	missense	E21	Het	PrD	D	3.971	Novel	UVS
168	RP	Panel 6	MERTK	c.845‐1G>A	—	splice	I5	Het	—	—	‐	Novel	P
			MERTK	c.1169T>A	p.(Val390Asp)	missense	E8	Het	PrD	D	1.547	Novel	LP
157	RP	Panel 6	RP1	c.4905_4906delGT	p.(Tyr1636Argfs*2)	deletion	E4	Het	—	—	3.619	Novel	LP
			RP1	c.6181delA	p.(Ile2061Serfs*12)	deletion	E4	Het	—	—	0.277	PMID:30027431	P
12	RP	Panel 1	RP2	c.409‐411delATT	p.(Ile137del)	deletion	E2	Hem	—	—	4.494	PMID:10937588	P
79	RP	Panel 1	RP2	c.353G>A	p.(Arg118His)	missense	E2	Hem	PrD	D	5.5	PMID:10937588	LP
15	RP	Panel 2	RPGR	c.2006G>A	p.(Trp669*)	nonsense	E15	Hem	—	—	1.007	Novel	LP
68	RP	WES	RPGR	c.2293delG	p.(Glu765Argfs*50)	deletion	E15	Hem	—	—	0.138	Novel	LP
176	RP	Panel 6	RPGR	c.818A>G	p.(Gln273Arg)	missense	E8	Hem	PrD	D	4.289	Novel	LP

Abbreviations: B, benign; Bietti, Bietti crystalline corneoretinal dystrophy; D, Deleterious; E, Exon; Hem, hemizygous; Het, heterozygous; Hom, homozygous; I, Intron; LP, Likely pathogenic; N, Neutral; P, pathogenic; PoD, possibly damaging; PrD, probably damaging; RP, retinitis pigmentosa; Usher, Usher syndrome; UVS, uncertain significance; WES, whole exome sequencing.

**Table 3:** Heterozygous variants with only one hit for autosomal recessive retinitis pigmentosa genes

No.ID	Disease	Panel	Gene	Nucleotide change	Amino acid change	Variant type	Exon/Intron	Hom/Het/Hem	Polyphen2	SIFT	PhyloP	Reference	ACMG
2	Usher	Panel 1	USH2A	c.9815C>T	p.(Pro3272Leu)	missense	E50	Het	PrD	D	5.593	PMID:18281613	LP
88	RP	Panel 1	USH2A	c.13465G>A	p.(Gly4489Ser)	missense	E63	Het	PrD	D	0.735	PMID:29641573	LP
166	Usher	Panel 6	USH2A	c.5309A>T	p.(Lys1770Ile)	missense	E27	Het	PrD	N	2.788	Novel	UVS
45	RP	Panel 1	EYS	c.6416G>A	p.(Cys2139Tyr)	missense	E31	Het	PrD	D	1.583	PMID:25753737	LP
77	RP	Panel 2	EYS	c.6416G>A	p.(Cys2139Tyr)	missense	E31	Het	PrD	D	1.583	PMID:25753737	LP
84	RP	WES	EYS	c.6557G>A	p.(Gly2186Glu)	missense	E32	Het	PoD	D	0.561	PMID:25356976	P
104	RP	Panel 1	EYS	c.9248G>A	p.(Gly3083Asp)	missense	E43	Het	PrD	N	2.306	PMID:27375351	LP
30	RP	Panel 1	CLRN1	c.407G>A	p.(Gly136Glu)	missense	E2	Het	PrD	D	1.197	PMID:27610647	LP
141	RP	Panel 5	CERKL	c.566delA	p.(Lys189Argfs*6)	deletion	E3	Het	—	—	2.619	Novel	LP
31	RP	Panel 1	RP1	c.1372A>T	p.(Arg458*)	nonsense	E4	Het	—	—	0.461	Novel	LP
73	RP	WES	CRB1	c.2222T>C	p.(Met741Thr)	missense	E7	Het	PoD	D	2.384	PMID:24535598	LP
111	RP	Panel 5	SLC7A14	c.524G>A	p.(Gly175Glu)	missense	E3	Het	PrD	D	5.625	Novel	UVS

Abbreviations: B, benign; Bietti, Bietti crystalline corneoretinal dystrophy; D, Deleterious; E, Exon; Hem, hemizygous; Het, heterozygous; Hom, homozygous; LP, Likely pathogenic; *N*, Neutral; P, pathogenic; PoD, possibly damaging; PrD, probably damaging; RP, retinitis pigmentosa; Usher, Usher syndrome; UVS, uncertain significance; WES, whole exome sequencing.

**Figure 2:**
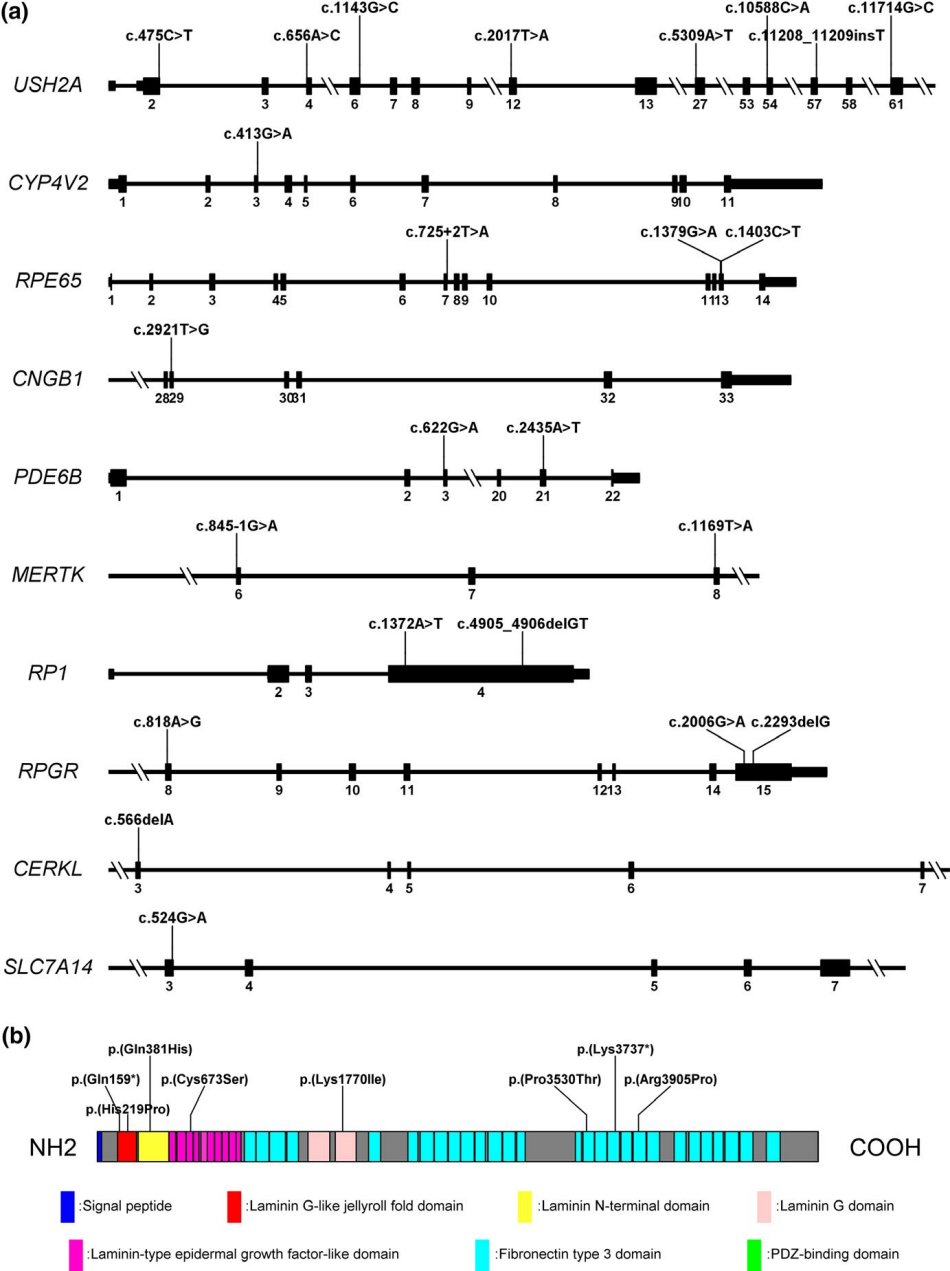
(a) Schematic representations of genomic structures of genes showing locations of novel variants. Numbers below diagram indicate corresponding exon numbers. Parts of exons are omitted. (b) Schematic representation of USH2A protein showing locations of novel variants. Notably, the PDZ‐binding domain in the last section of the schematic representation in green is difficult to identify because it constitutes two amino acids

**Figure 3:**
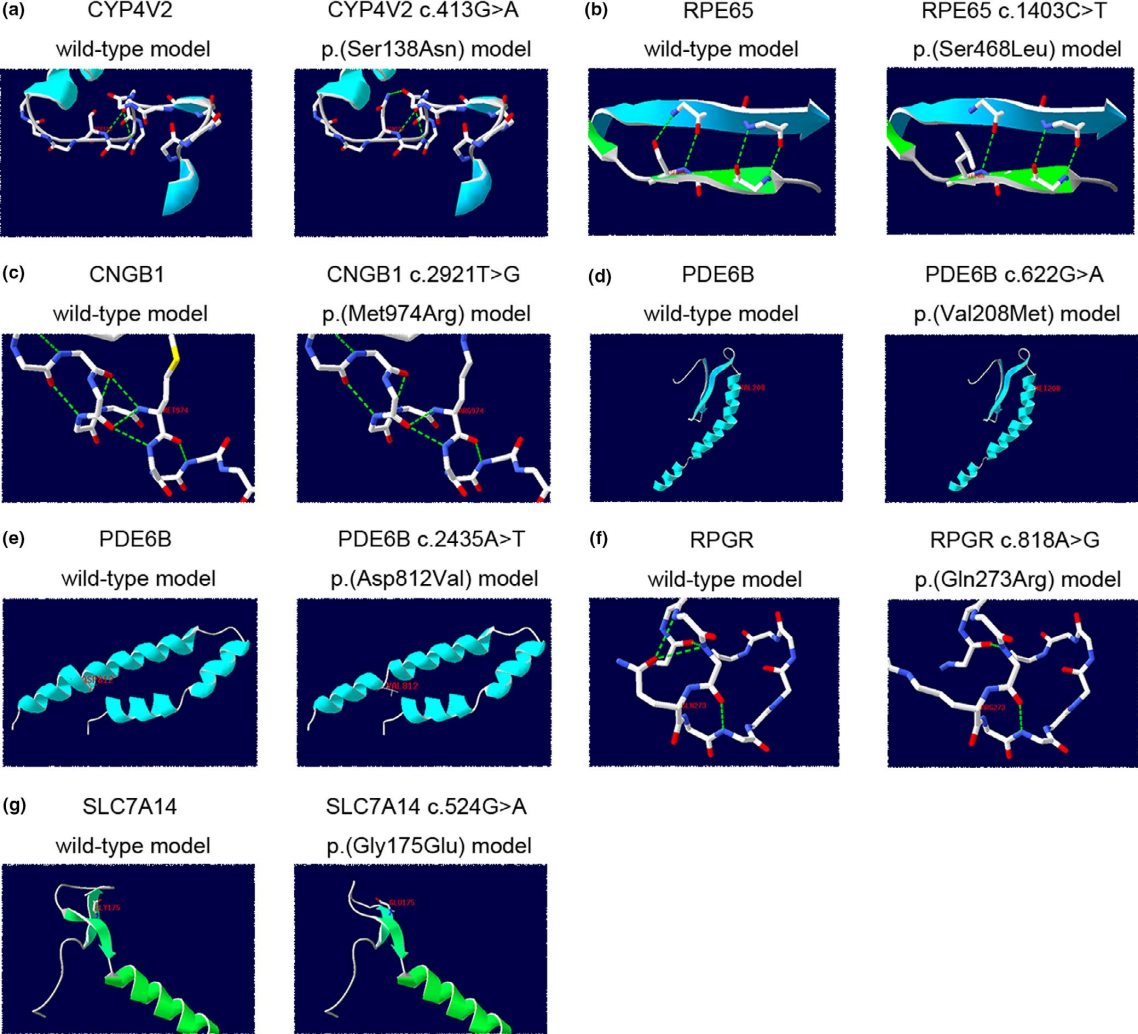
Topology and molecular models of seven novel variants. (a) CYP4V2 protein molecular alteration caused by CYP4V2 variant c.413G>A, p.(Ser138Asn). These models were predicted using 6c94.1. Compared to the wild‐type model, serine is replaced by aspartic acid, which creates H‐bonds (green dash line) between residues in the mutant model. (b) RPE65 protein molecular alteration caused by RPE65 variant c.1403C>T p.(Ser468Leu). These models were predicted using 4f30.1. Compared to the wild‐type model, the number of H‐bonds (green dash line) between residues in the mutant model markedly decreased. (c) CNGB1 protein molecular alteration caused by CNGB1 variant c.2921T>G p.(Met974Arg). These models were predicted using 5h3o.1. Compared to the wild‐type model, the number of H‐bonds (green dash line) between residues in the mutant model markedly decreased. (d) PDE6B protein molecular alteration caused by PDE6B variant c.622G>A p.(Val208Met). These models were predicted using 6mzb.1. There was no major difference between the wild‐type and mutant models. (e) PDE6B protein molecular alteration caused by PDE6B variant c.2435A>T, p.(Asp812Val). These models were predicted using 6mzb.1. Compared to the wild‐type model, the last helix is divided in the mutant model. (f) RPGR protein molecular alteration caused by RPGR variant c.818A>G, p.(Gln273Arg). These models were predicted using 4jhn.1. Compared to the wild‐type model, the number of H‐bonds (green dash line) between residues in the mutant model markedly decreased. (g) SLC7A14 protein molecular alteration caused by SLC7A14 variant c.524G>A, p.(Gly175Glu). These models were predicted using 6f34.1. Compared to the wild‐type model, glycine is replaced by glutamic acid, which changes the direction of beta strand folding in the mutant model

In total, we identified 67 potential pathogenic gene variants; these included 38 missense variants (52.2%), 10 nonsense variants (16.4%), 1 small indel variant (1.5%), 10 small deletion variants (14.9%), 2 small insertion variants (3.0%), and 6 splice variants (9.0%). The proportions of all types of variants are shown in Figure 1b. Of these 67 potential pathogenic variants, 24 were novel. The pedigrees of the probands with novel variants are shown in Figure S3; the sequencing chromatographs of novel variants and corresponding wild‐type alleles are shown in Figure S4. Schematic representations of the genomic structures of genes with novel variants are shown in Figure 2a. The eight *USH2A* novel variants were distributed irregularly among the exons of *USH2A*; these variants presumably affect specific domains of the USH2A protein (Figure 2b). The topology and molecular models of seven novel variants showed molecular alterations in proteins caused by mutations, except in the *PDE6B* variant c.622G>A, p.(Val208Met) (Figure 3).

## 4 DISCUSSION

Despite the advent of the personalized medicine era, traditional sequencing has not been able to achieve precise genetic diagnosis (Neveling et al., 2013). NGS technology is regarded as a powerful and effective tool for the detection of pathogenic gene variants underlying genetic RP (Gilissen, Hoischen, Brunner, & Veltman, 2011; Lovric et al., 2014; Riera et al., 2017; Wang et al., 2019). In this study, we used NGS technology, bioinformatics prediction, Sanger sequencing validation, and available family member segregation; we identified 43 families (56.6%) with disease‐causing gene variants, whereas the detection rates were 63.5%, 50%, and 58% in previous studies (Huang et al., 2018; Neveling et al., 2012; Xu et al., 2015). The detection rate of gene variants in patients with RP was higher with targeted panel sequencing and whole exome sequencing than with microarray genotyping (Avila‐Fernandez et al., 2010; Blanco‐Kelly et al., 2012), targeted‐capture sequencing (Fu et al., 2013; Wang et al., 2014), or individual gene sequencing (Sweeney, McGee, Berson, & Dryja, 2007). In the present study, the detection rates of Usher syndrome, Bardet–Biedl syndrome, and Bietti crystalline corneoretinal dystrophy were 17.1% (13 probands), 1.3% (1 proband), and 6.6% (5 probands), respectively. In these targeted panels, panel 5 was the most informative in Chinese patients with RP due to its relatively high detection rate (71.4%). The detection rate of novel variants among all identified variants was 35.8%, whereas the detection rates were 72.7% and 67% in previous studies (Huang et al., 2018; Xu et al., 2014). The higher novel detection rate observed in the prior studies was potentially because probands without identified gene variants were enrolled in those studies. The detection rate of variants in *USH2A,* the causative gene most frequently identified in this study, was 19.7% (15 probands). Among families with nonsyndromic RP, variants in *USH2A* were identified in 8.1% (five probands), which was higher than the rate in a study of North American families (7%) (Seyedahmadi, Rivolta, Keene, Berson, & Dryja, 2004) and the rate in a study of Spanish families (7%) (Avila‐Fernandez et al., 2010). Variants c.8559‐2A>G and c.11156G>A in *USH2A* were recurrent, as they were found in five and four probands, respectively. We presume that these variants are founder variants.

In the study, we did not find a disease‐causing variant in 21 families (27.6%), whereas we found only one heterozygous variant of arRP genes in 12 families (15.8%). Possible reasons for these results are as follows. First, targeted panels sequencing and WES cannot capture variants in the noncoding regions of corresponding genes, nor can they detect variants comprising gross deletions, gross insertions, or complex rearrangements (Broadgate, Yu, Downes, & Halford, 2017). Second, the sequencing depth of coverage was insufficient to accurately call all variants, especially those located in regions with high GC content. Third, variants of novel genes in patients with RP may have been filtered out in raw data analysis (Daiger, Sullivan, & Bowne, 2013). Fourth, other mild and moderate systemic clinical manifestations of syndromic RP may have been neglected (Xu et al., 2014). Fifth, small indel, large structural, copy number, or duplication variants in patients with Usher syndrome are not readily identified with NGS technology (Bonnet et al., 2016; O'Donnell‐Luria & Miller, 2016). Whole genome sequencing may be a comprehensive alternative strategy because it partially resolves these problems (Carrigan et al., 2016).

In this study, we also detected two novel hemizygous *RPGR* variants c.2006G>A, p.(Trp669*) and c.818A>G, p.(Gln273Arg). These variants did not segregate with the disease in family Family 15 and Family 176. Both of the probands’ biological parents exhibited wild‐type genotypes without histories of bone marrow transplant surgery. The lack of segregation was possibly because the variants were de novo or because the probands’ mothers exhibited chimerism. Other examinations (e.g., high‐depth DNA sequencing of oral mucosa and urinary sediment for somatic cell chimerism, or of an ovum for gonad chimerism) are needed to definitively determine the statuses of the probands’ mothers.

This study identified the gene variants in a cohort of Chinese probands with RP; however, there were some limitations. Some panels did not allow analysis of all RP genes. Furthermore, some families could not undergo segregation analysis. We plan to perform WES or whole genome sequencing to capture more genes and include patients in future research.

In conclusion, we enrolled a cohort of 76 families who exhibited RP. We identified 43 families (56.58%) with disease‐causing variants in 15 genes and 12 families (15.79%) with only one heterozygous variant in arRP genes. We also detected 67 potential pathogenic gene variants, of which 24 have not been previously described. These results will provide useful data for clinicians to make accurate genetic diagnosis, prognosis estimation, and genetic counseling; moreover, they will provide further support for researchers to explore RP pathogenesis.

## CONFLICT OF INTEREST

The authors declare that they have no conflict of interest with regard to this work.

## Data Availability

They are available on special request.
