# Supplementary figures and images for: Application of targeted panel sequencing and whole exome sequencing for 76 Chinese families with retinitis pigmentosa
Source: Mol Genet Genomic Med. 2020 Jan 20;8(3):e1131. doi: 10.1002/mgg3.1131 (PMC7057118; doi:10.1002/mgg3.1131)

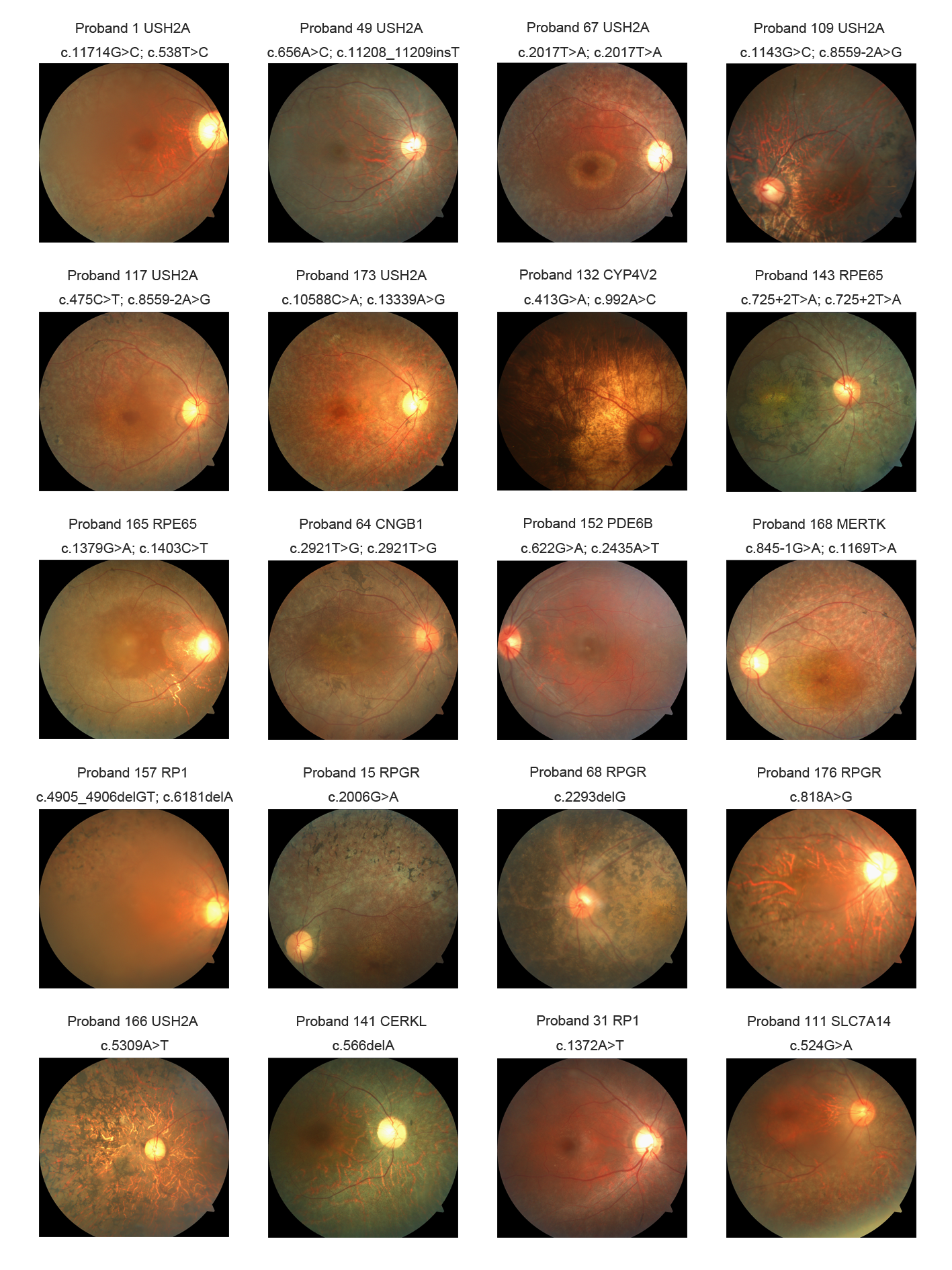

Supplement: Supplementary file 1 [file MGG3-8-e1131-s001.tif]

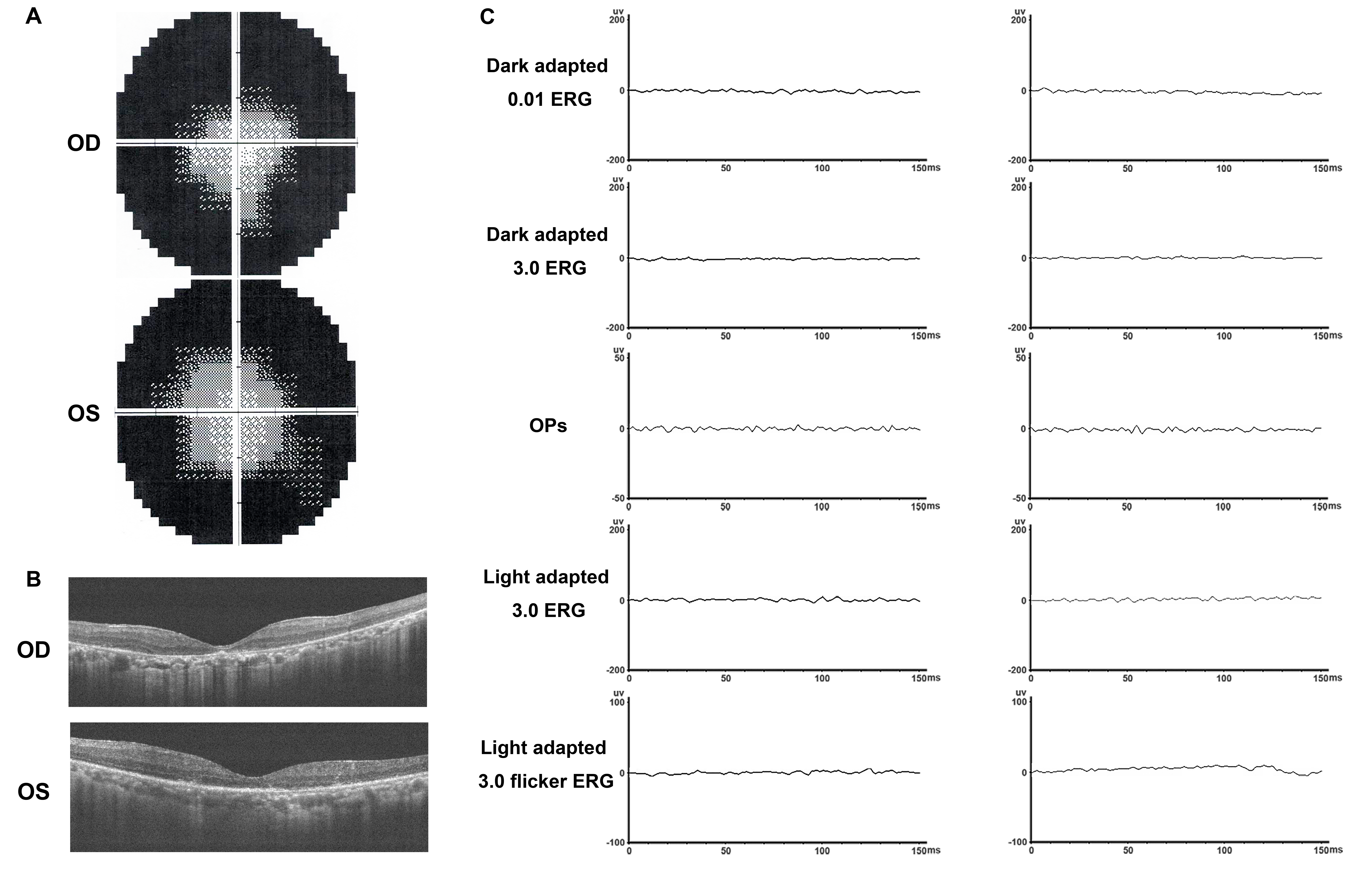

Supplement: Supplementary file 2 [file MGG3-8-e1131-s002.tif]

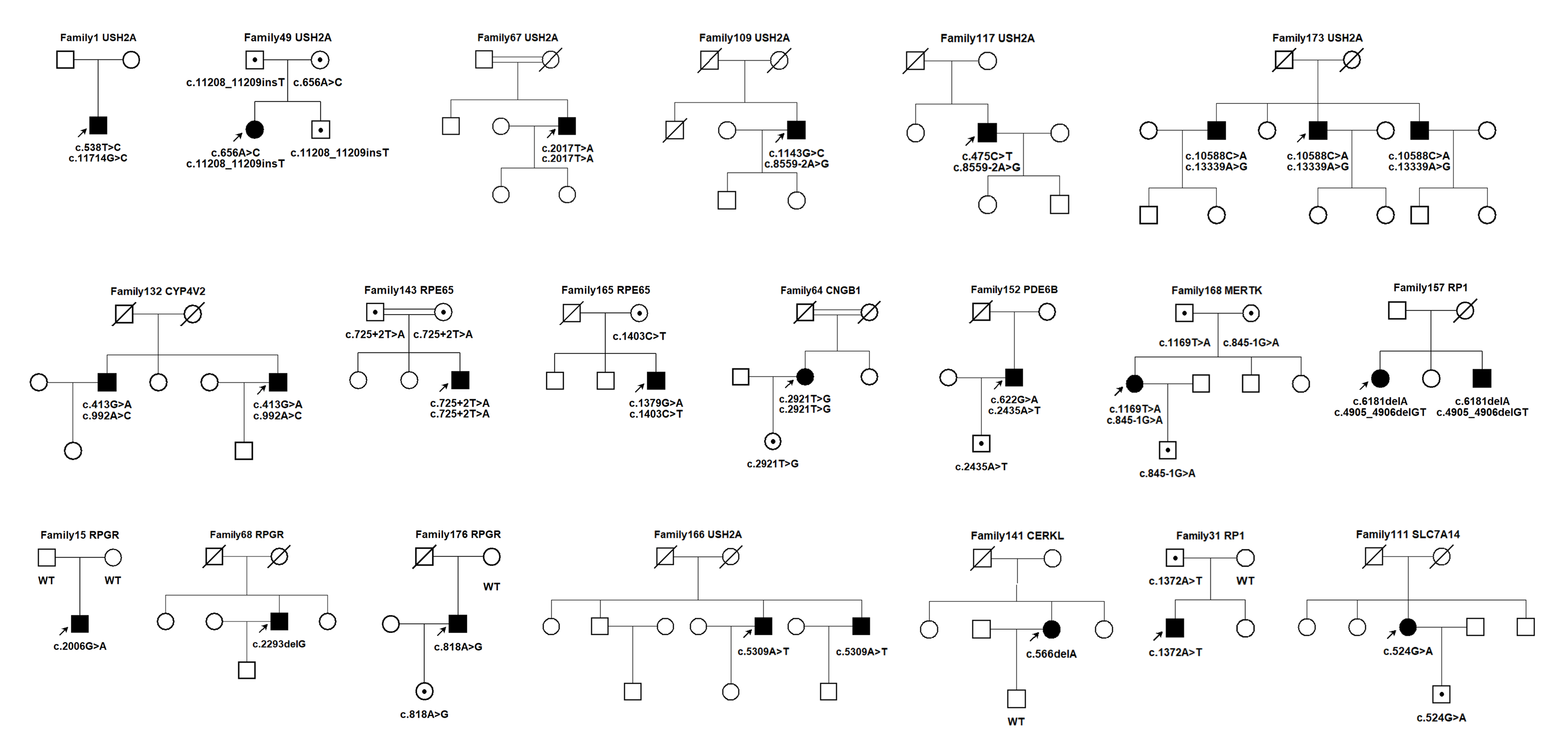

Supplement: Supplementary file 3 [file MGG3-8-e1131-s003.tif]

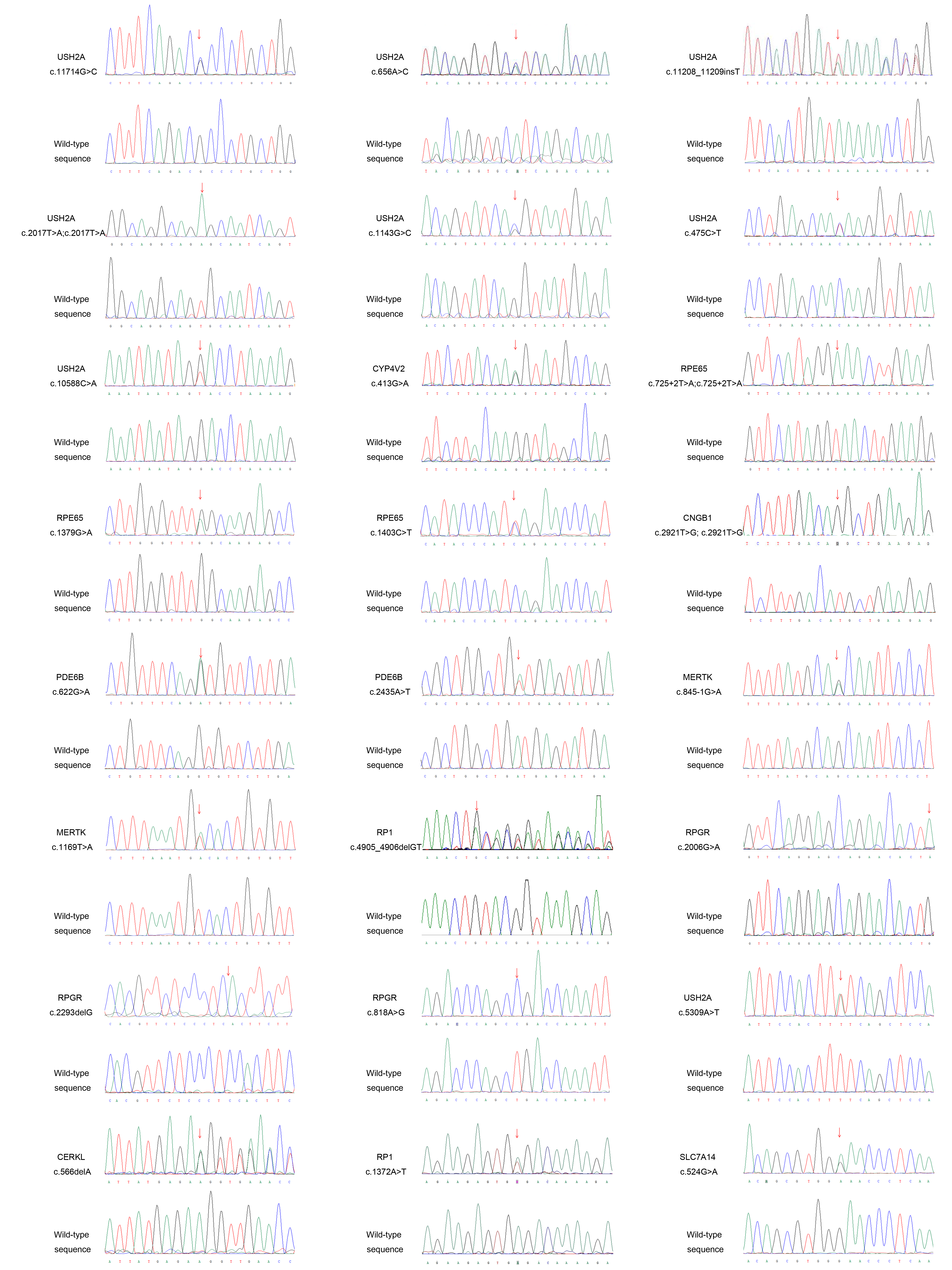

Supplement: Supplementary file 4 [file MGG3-8-e1131-s004.tif]
