# Supplementary material for: Application of targeted panel sequencing and whole exome sequencing for 76 Chinese families with retinitis pigmentosa
Source: Mol Genet Genomic Med. 2020 Jan 20;8(3):e1131. doi: 10.1002/mgg3.1131 (PMC7057118; doi:10.1002/mgg3.1131)
Supplement: Supplementary file 5 [file MGG3-8-e1131-s005.docx]

Supplementary Table 2. Primers used in Sanger sequencing

| **Gene** | **Nucleotide change** | **Location** | **Primer** | **Amplicon (bp)** | |
| --- | --- | --- | --- | --- | --- |
| *RHO* | c.1045T>C | 3:129252559-129252559 | TGGATTTGAGTGGATGGGGC | | 597 |
|  |  |  | TGTGCCCCATTCTGTGCTAG | |  |
| *RHO* | c.1040C>T | 3:129252554-129252554 | TGGATTTGAGTGGATGGGGC | | 597 |
|  |  |  | TGTGCCCCATTCTGTGCTAG | |  |
| *PRPF31* | c.220C>T | 19:54621995-54621995 | GATCGAGGATGTGCAGGAGG | | 551 |
|  |  |  | GGCAAAAACCCTGTAACCGG | |  |
| *USH2A* | c.538T>C | 1:216591969-216591969 | ACAGCAATTTTGGGGGAGGA | | 911 |
|  |  |  | GCCAGTTGATGATGGGGTGA | |  |
| *USH2A* | c.11714G>C | 1:215901724-215901724 | CACTGTGAAAGCATGCACGG | | 528 |
|  |  |  | TGGAGTTAGGCCACTGGAGT | |  |
| *USH2A* | c.142_143insGA | 1:216595536-216595536 | TGAGGCCTGCTGAGAAAAGG | | 589 |
|  |  |  | GCCTGGGATGAGCTTCAGTT | |  |
| *USH2A* | c.4165delG | 1:216369980-216369981 | CCTGTGAAAACGCCATGGGA | | 599 |
|  |  |  | CAGCTGCATTGGATTGGCTC | |  |
| *USH2A* | c.11156G>A | 1:215933077-215933077 | TGCTAGTTCAAGGATGCGTGT | | 501 |
|  |  |  | CTTGCCAGGAGTTTGGGTGA | |  |
| *USH2A* | c.4645C>T | 1:216270538-216270538 | ACCTCAGTACCAGGCACCTA | | 575 |
|  |  |  | AGCAAGACCCTCTGTGTTTCT | |  |
| *USH2A* | c.1397G>T | 1:216496969-216496969 | TGGTGATTTCGTCCACTGCA | | 589 |
|  |  |  | ACCACCAACTTCCTTGAGGC | |  |
| *USH2A* | c.656A>C | 1:216538423-216538423 | ACCAACACCACTGAACCCTT | | 582 |
|  |  |  | TACCTGTGAACACCCTGGGA | |  |
| *USH2A* | c.11208_11209insT | 1:215933024-215933024 | TGCTAGTTCAAGGATGCGTGT | | 502 |
|  |  |  | CTTGCCAGGAGTTTGGGTGA | |  |
| *USH2A* | c.2017T>A | 1:216424395-216424395 | CCAGCCTGTCTTGAGCAAAG | | 491 |
|  |  |  | TCTGTATATTCCCCCTGCTGA | |  |
| *USH2A* | c.8559-2A>G | 1:216051224-216051224 | AGCTGGGTGATTAATGCCACA | | 582 |
|  |  |  | CTTTGTGCGAAACTGGCAGT | |  |
| *USH2A* | c.1143G>C | 1:216498647-216498647 | GGGTCTAATAGGGTGGGCCT | | 515 |
|  |  |  | TGATGCAGGAGACACAGCTG | |  |
| *USH2A* | c.2802T>G | 1:216419934-216419934 | TCCGATCGGCTGAGTTTTATCT | | 510 |
|  |  |  | TGTCTGCCTTGCAACTGTGA | |  |
| *USH2A* | c.4616C>T | 1:216348605-216348605 | AACGGGGTGCATACTGAGAA | | 600 |
|  |  |  | GGCCACCTCTGGTTAAAGGA | |  |
| *USH2A* | c.475C>T | 1:216595204-216595204 | CCAAAGGTCGCGCTAAGCTA | | 524 |
|  |  |  | CAGTTCTGTACCCAGCGGTT | |  |
| *USH2A* | c.14426C>T | 1:215822026-215822026 | ACACCATTGGGGAACATGGG | | 548 |
|  |  |  | CACTTTGTGTGTTCCGTGCC | |  |
| *USH2A* | c.9958G>T | 1:215972249-215972249 | ATTGGCACAGTGTCTGTTAGT | | 541 |
|  |  |  | GTGCTGGGAGGCTTCATGAT | |  |
| *USH2A* | c.9815C>T | 1:215972392-215972392 | TAGACCTGGGCCCCTTACC | | 501 |
|  |  |  | GTCCATGTCACCAGACCTGA | |  |
| *USH2A* | c.13465G>A | 1:215847788-215847788 | AGGGGCTGGTTCGATCTTTG | | 544 |
|  |  |  | ATCACAACTCTGGAGGCTGC | |  |
| *USH2A* | c.10588C>A | 1:215955536-215955536 | TACCTTGCTACTGGTGGCAC | | 545 |
|  |  |  | TCACTTTGTGGTGTAGTCCTCG | |  |
| *USH2A* | c.13339A>G | 1:215847914-215847914 | AGGGGCTGGTTCGATCTTTG | | 544 |
|  |  |  | ATCACAACTCTGGAGGCTGC | |  |
| *USH2A* | c.5309A>T | 1:216251694-216251694 | AGTTCAGCAGTTCCTGTGGG | | 572 |
|  |  |  | TCAGTCACTTATGCAGGGGT | |  |
| *CLRN1* | c.253+6T>C | 3:150690237-150690237 | GAGCCCATGACCTTGGTTCT | | 574 |
|  |  |  | CTCGGAGTTGTGACAGCCTT | |  |
| *CLRN1* | c.407G>A | 3:150659395-150659395 | CCCTACTGTTGAGCAAGTGTG | | 682 |
|  |  |  | ACCGAGAAAGGTCATTAAAGGCT | |  |
| *BBS2* | c.563delT | 16:56543917-56543918 | TACAGCTCTGTCTGACCCCC | | 509 |
|  |  |  | GCTGATCCAGGGAGCCTTTT | |  |
| *BBS2* | c.1237C>T | 16:56534926-56534926 | CCCAAGAATCCACTGGGCAT | | 505 |
|  |  |  | CTCTGTCTTGGCTCACTGCA | |  |
| *CYP4V2* | c.802-6_810delATACAGGTCATCGCT | 4:187122304-187122319 | GCAGCAGAAATCGCAAGCAT | | 591 |
|  |  |  | GCCTGTTCCCTTCGTCATCA | |  |
| *CYP4V2* | c.1199G>A | 4:187130127-187130127 | CCCCACTGCTCTTTCAGGTC | | 560 |
|  |  |  | AAGGCTGTTGCATATGGGCA | |  |
| *CYP4V2* | c.1091-2A>G | 4:187130017-187130017 | TCCACGTGTTCTTTGATGGG | | 559 |
|  |  |  | TGAGACCGGAGCAAGGTAGA | |  |
| *CYP4V2* | c.802-8_810delTCATACAGGTCATCGCG/insGC | 4:187122302-187122319 | GCAGCAGAAATCGCAAGCAT | | 591 |
|  |  |  | GCCTGTTCCCTTCGTCATCA | |  |
| *CYP4V2* | c.413G>A | 4:187117242-187117242 | TCCTGGTTTATGGCGGGAAC | | 597 |
|  |  |  | TCTTCCTGAACACTGGCGAC | |  |
| *CYP4V2* | c.992A>C | 4:187126358-187126358 | CATGAACCTCCGCTTTGCAG | | 572 |
|  |  |  | CGACCCATGACTCAAAGCCT | |  |
| *EYS* | c.8545C>T | 6:64431382-64431382 | ATTTCCAGCCCAATCTGGCA | | 572 |
|  |  |  | CCAACTTGGCCAGAAACAGC | |  |
| *EYS* | c.5644+5G>A | 6:65300111-65300111 | GCAGTATAGCTGTCTCCAACCT | | 557 |
|  |  |  | CCACCATTGACAGGCTCAGT | |  |
| *EYS* | c.2953_2961delACTGATGGA | 6:65596620-65596629 | TTTGCCCTGTTTGCATCTGG | | 921 |
|  |  |  | TGCTAAGTATACAGTTTCGATCAGT | |  |
| *EYS* | c.8805C>A | 6:64431122-64431122 | TGGAGACCAATTGCCAGAAA | | 510 |
|  |  |  | TCAAGGCTGTATCCGACAAGT | |  |
| *EYS* | c.4955C>A | 6:65300805-65300805 | ACCTCAGTGGGTCCCATAGT | | 560 |
|  |  |  | ACTGAGGCTTCAAGCAACCA | |  |
| *EYS* | c.6557G>A | 6:64791763-64791763 | TGTCTTGTCAGCTCAGATCCTG | | 583 |
|  |  |  | TCAGTCTTTTCCTCTGTACTGGT | |  |
| *EYS* | c.9209T>C | 6:64430718-64430718 | ACAGTTGATTCCCCGTAAGCA | | 506 |
|  |  |  | TTTCTGGCAATTGGTCTCCA | |  |
| *EYS* | c.3489T>A | 6:65336093-65336093 | AGTACTTGCTGTGTGCCAGG | | 571 |
|  |  |  | GGCTCCCAGCTACATGTTGT | |  |
| *EYS* | c.6416G>A | 6:64940493-64940493 | TGTCTCTGTGGAAACAAAGGA | | 539 |
|  |  |  | TCTCCAACAGCCTCCTTTGT | |  |
| *EYS* | c.9248G>A | 6:64430679-64430679 | ACAGTTGATTCCCCGTAAGCA | | 506 |
|  |  |  | TTTCTGGCAATTGGTCTCCA | |  |
| *RPE65* | c.131G>A | 1:68912507-68912507 | ACTGGCCCAGGTACATTGTG | | 512 |
|  |  |  | GGGCATCTCCACATTTCCCA | |  |
| *RPE65* | c.725+2T>A | 1:68905242-68905242 | AGGGCTGATTCTCAAGCCAC | | 569 |
|  |  |  | AACCCACCTCCAGCTATTGC | |  |
| *RPE65* | c.1379G>A | 1: 68896819-68896819 | GTCTTCGTCAGGGAAGGCAA | | 577 |
|  |  |  | TGCGTATGGACTTGGCTTGA | |  |
| *RPE65* | c.1403C>T | 1: 68896795-68896795 | GTCTTCGTCAGGGAAGGCAA | | 577 |
|  |  |  | TGCGTATGGACTTGGCTTGA | |  |
| *CNGA1* | c.829G>A | 4:47942822-47942822 | CTTGTACCCTTCAACCCACTTATTC | | 554 |
|  |  |  | ATCTAAGCCTATGAGTTTATACATCGTG | |  |
| *CNGA1* | c.472delC | 4:47951883-47951884 | CACTATGTTTGTTCAAGACACTGGC | | 722 |
|  |  |  | GACTTCTCCCTCTGCCCAATAC | |  |
| *CNGB1* | c.2921T>G | 16:57935311-57935311 | CTTGGGTCTGGTCAGGTCAC | | 503 |
|  |  |  | GCCGGTATGTATGTCTGGGG | |  |
| *MERTK* | c.845-1G>A | 2:112725713-112725713 | TTTGTTTGGTAGCTGTAGCCTGTCA | | 448 |
|  |  |  | CTCATGATGTTCTCCCTTGGAAAC | |  |
| *MERTK* | c.1169T>A | 2:112740443-112740443 | ACACTTGAAAACCCAGATGAGAATAC | | 513 |
|  |  |  | TCAGGTGATAACAGAGGATGAATTCT | |  |
| *PDE6B* | c.622G>A | 4:629669-629669 | AGAGAGATAGCTTGCGTGCC | | 597 |
|  |  |  | CCTGCTCACTCGTCACTCAT | |  |
| *PDE6B* | c.2435A>T | 4:661727-661727 | GGACTGGTGGTGACTTCTCG | | 502 |
|  |  |  | GGGGTTCACTGGCTAGGAAC | |  |
| *RP1* | c.4905_4906delGT | 8:55541346-55541348 | TGGTCAGACTATCGGCCTGA | | 542 |
|  |  |  | GATGTGGTGTCTGCATTGCC | |  |
| *RP1* | c.6181delA | 8:55542620-55542621 | AGGAAGACCGAGGATTTGCA | | 555 |
|  |  |  | AGGAGGTCTCAACTTGGCAA | |  |
| *RP1* | c.1372A>T | 8:55537814-55537814 | GCTGAAACTTGCAGTTCTGCT | | 596 |
|  |  |  | AAATGGGTTGCATCTGCTGA | |  |
| *CERKL* | c.566delA | 2:182438526-182438527 | AGGGGTCAGAGTCAGAACGA | | 548 |
|  |  |  | AGGCTTTCCAAACAGACCGA | |  |
| *CRB1* | c.2222T>C | 1:197396677-197396677 | TGCTTGTGTGCATGTGTGTG | | 506 |
|  |  |  | CTTGCTTGTCAGGTAGGCCA | |  |
| *SLC7A14* | c.524G>A | 3:170218915-170218915 | GTCACTTGCCCCACTGTAGA | | 533 |
|  |  |  | TATGTCTGTCCCTTGCAGGC | |  |
| *RP2* | c.409-411delATT | X:46712805-46712806 | AGTGTCACAGAGGCTACCCT | | 542 |
|  |  |  | ACACGAAATTGTTGGCAGGC | |  |
| *RP2* | c.353G>A | X:46713161-46713161 | GACGGTAGCAGGACAACAGT | | 523 |
|  |  |  | CTGTCTCTGACCCCGGGATA | |  |
| *RPGR* | c.2006G>A | X:38146246-38146246 | CCCTGCTCCCTCTCCTTTTG | | 585 |
|  |  |  | TGATGGATTCCAGCAGCCTG | |  |
| *RPGR* | c.2293delG | X:38145958-38145959 | CTCCTTTCCCCTCCTCTACT | | 547 |
|  |  |  | TCAGTGGGAGAAGCAGAGGA | |  |
| *RPGR* | c.818A>G | X:38164004-38164004 | CTTCTGACATCATCGGCCTATTG | | 518 |
|  |  |  | TTTTCCCCAGAGGCACTTAACCT | |  |
