# Supplementary material for: Application of targeted panel sequencing and whole exome sequencing for 76 Chinese families with retinitis pigmentosa
Source: Mol Genet Genomic Med. 2020 Jan 20;8(3):e1131. doi: 10.1002/mgg3.1131 (PMC7057118; doi:10.1002/mgg3.1131)
Supplement: Supplementary file 6 [file MGG3-8-e1131-s006.docx]

Supplementary Table 3. Genomic information of genes responsible for retinitis pigmentosa in the families in this study

| **Disease Category** | **Gene** | **gDNA** | **mRNA** | **Protein** |
| --- | --- | --- | --- | --- |
| adRP | *RHO* | NC_000003.11 | NM_000539.3 | NP_000530.1 |
|  | *PRPF31* | NC_000019.9 | NM_015629.3 | NP_056444.3 |
| arRP | *USH2A* | NC_000001.10 | NM_206933.2 | NP_996816.2 |
|  | *CLRN1* | NC_000003.11 | NM_174878.2 | NP_777367.1 |
|  | *BBS2* | NC_000016.9 | NM_031885.3 | NP_114091.3 |
|  | *CYP4V2* | NC_000004.11 | NM_207352.3 | NP_997235.3 |
|  | *EYS* | NC_000006.11 | NM_001142800.1 | NP_001136272.1 |
|  | *RPE65* | NC_000001.10 | NM_000329.2 | NP_000320.1 |
|  | *CNGA1* | NC_000004.11 | NM_001142564.1 | NP_001136036.1 |
|  | *CNGB1* | NC_000016.9 | NM_001297.4 | NP_001288.3 |
|  | *PDE6B* | NC_000004.12 | NM_000283.3 | NP_000274.2 |
|  | *MERTK* | NC_000002.11 | NM_006343.2 | NP_006334.2 |
|  | *RP1* | NC_000008.10 | NM_006269.1 | NP_006260.1 |
|  | *CERKL* | NC_000002.11 | NM_001030311.2 | NP_001025482.1 |
|  | *CRB1* | NC_000001.10 | NM_201253.2 | NP_957705.1 |
|  | *SLC7A14* | NC_000003.11 | NM_020949.2 | NP_066000.2 |
| xlRP | *RP2* | NC_000023.10 | NM_006915.2 | NP_008846.2 |
|  | *RPGR* | NC_000023.10 | NM_001034853.1 | NP_001030025.1 |

Note: adRP=autosomal dominate retinitis pigmentosa; arRP=autosomal recessive retinitis pigmentosa; and xlRP=X-linked retinitis pigmentosa.
