# Supplementary material for: Application of targeted panel sequencing and whole exome sequencing for 76 Chinese families with retinitis pigmentosa
Source: Mol Genet Genomic Med. 2020 Jan 20;8(3):e1131. doi: 10.1002/mgg3.1131 (PMC7057118; doi:10.1002/mgg3.1131)
Supplement: Supplementary file 7 [file MGG3-8-e1131-s007.docx]

Supplementary Text 1: Genes for targeted next-generation sequencing in the panels

Panel 1. sequencing platform: Ion Torrent PGM

*FOXE3, RPE65, ABCA4, PRPF3, MYOC, CRB1, USH2A, CYP1B1, CNGA3, CERKL, SAG, GNAT1, HESX1, RHO, CLRN1, SOX2, PDE6B, CNGA1, PITX2, GPR98, PDE6A, GRM6, FOXC1, GUCA1A, PRPH2, EYS, ELOVL4, TSPAN12, IMPDH1, RP1, CNGB3, OPTN, PCDH15, CDH23, USH1C, DCDC1, ELP4, PAX6, WT1, BEST1, CABP4, LRP5, MYO7A, FZD4, RDH5, CEP290, RPGRIP1, OTX2, SIX6, VSX2, TRPM1, NR2E3, STRA6, RLBP1, PRPF8, AIPL1, GUCY2D, DFNB3, RAX, CRX, PRPF31, XLRS1, BCOR, NYX, NDP, RP2, CACNA1F, CHM, FRMD7, RDS*

Panel 2. sequencing platform: Ion Torrent PGM

*CDH3, RGS9, OPN1SW, CDHR1, LTBP2, GPR98, GALT, EYS, PDE6A, NBAS, BFSP1, CNBP, OPN1MW, INPP5E, ABCC6, MFSD6L, VCAN, RPE65, ADAMTS18, COL9A1, PANK2, CRYBA1, MYO7A, NHS, PITX2, CHRDL1, ROM1, ALDH1A3, BBS2, RLBP1, CNGB1, MITF, RP1, CDH23, ZEB1, DTHD1, BFSP2, PRPF6, GRM6, PRDM5, MIR184, NPHP4, CRYBB3, PEX2, IMPG2, POLG2, CC2D2A, GALK1, HARS, SIX6, SDCCAG8, GPR143, ZNF469, FSCN2, TSPAN12, CLRN1, HOXA1, GPR179, CERKL, PIKFYVE, FLVCR1, INVS, CRX, FAM161A, NTF4, TEAD1, VIM, AKR1E2, BBS9, CRYGD, PHOX2A, VSX2, HMX1, NMNAT1, CHM, CRYAA, KIF21A, BEST1, RAB3GAP1, PITPNM3, OPTN, CABP4, PDE6H, CRYGC, RBP4, IGBP1, UBIAD1, MAF, CEP290, TIMP3, CA4, DMD, CIB2, RPGR, PRPH2, RRM2B, RP1L1, WDR36, BBS12, EPHA2, ADGRA3, SAG, CHD7, PEX7, IMPDH1, 　PHGDH, MIP, ADAM9, TULP1, RPGRIP1, OTX2, HSF4, VAX1, GRK1, AGK, SEMA4A, PITX3, USH2A, PAX6, PLA2G5, UNC119, LRP5, ADAMTSL4, ZNF513, GUCY2D, NPHP1, CNNM4, RPGRIP1L, GJA3, ATXN7, GDF6, SLC25A4, GNAT2, BBS7, NRL, KCNV2, CNGA3, RBP3, RAX2, HCCS, FYCO1, TDRD7, MAK, ZNF644, SLC16A12, ADAMTS10, CEP164, RS1, NR2E3, PCDH15, EMC1, FRMD7, GCNT2, C10orf2, PRPF31, SNRNP200, BMP4, SETX, RAB3GAP2, PDE6C, CACNA2D4, FOXC1, ACBD5, ALMS1, CYP27A1, CRYAB, DCN, LIM2, COL11A1, TTC8, KIF11, BBS4, GUCA1A, COL8A2, RAB18, TENM3, LCA5, PRCD, GNAT1, APOA1, IFT140, AIPL1, C2orf71, PRPF8, RGR, MKS1, KRT12, CRYBB2, MYH9, TRPM1, TTPA, RIMS1, COL2A1, LRIT3, DFNB31, PDZD7, RDH12, DHDDS, FZD4, TUBB3, C1QTNF5, CACNA1F, TMEM126A, CHN1, CRYBB1, TACSTD2, WDR19, SIL1, LRAT, PAX2, POLG, TMEM237, HDAC8, CNGB3, MYOC, GUCA1B, CSAD, POMT1, MFN2, BCOR, HMCN1, CRYGB, CRYBA4, KERA, RP2, FOXE3, PHYH, KIAA1549, RYR1, CHST6, TREX1, C12orf57, LEPREL1, PLOD3, TGFBI, SLC4A11, IGFBP7, GJA8, ZNF423, C21orf2, NDP, OFD1, WDPCP, KRT3, ABHD12, BBS1, IQCB1, SMOC1, MERTK, BBS10, RD3, JAG1, CRB1, IDH3B, DMPK, USH1G, RAX, OCRL, PDE6B, OPN1LW, RGS9BP, RP9, NPHP3, TRIM32, USH1C, SPATA7, STRA6, PDE6G, PEX1, CLN3, CYP51A1, PRSS56, CHMP4B, LZTFL1, KLHL7, PROM1, C8orf37, ROBO3, SLC24A1, CTDP1, ELOVL4, RB1, SOX2, VSX1, MTTP, CYP1B1, WRN, CNGA1, RDH5, KCNJ13, NYX, GNPTG, MKKS, AHI1, B3GALTL, TOPORS, RHO, CRYGS, PRPF3, CAPN5, MFRP, EFEMP1, ACO2, TIMM8A, ARL6, WFS1, IQSEC2, GDF3, PGK1, RNLS, CYP4V2, ABCA4, OAT, OPA3, BBS5, OPA1*

Panel 3. sequencing platform: HiSeq

*RP1, RP2, RPGR, RHO, RP9, IMPDH1, PRPF31, CRB1, PRPF8, TULP1, CA4, PRPF3, ABCA4, RPE65, OFD1, EYS, CERKL, NRL, FAM161A, FSCN2, TOPORS, PRCD, NR2E3, MERTK, USH2A, SNRNP200, SEMA4A, PDE6B, PROM1, KLHL7, PDE6A, RGR, CNGB1, IDH3B, SAG, GUCA1B, CNGA1, BEST1, TTC8, RDH12, C2orf71, ARL6, IMPG2, PDE6G, ZNF513, DHDDS, PRPF6, CLRN1, MAK, C8orf37, CDHR1, RBP3, NEK2, IFT172, ARL2BP, NYX, GRM6, TRPM1, SLC24A1, GNAT1, GPR179, LRIT3, CACNA1F, CABP4, GRK1, CYP4V2, SPATA7, AIPL1, LRAT, ABHD12, FLVCR1, RLBP1, NDP, GUCY2D, LCA5, CEP290, RD3, RPGRIP1*

Panel 4. sequencing platform: HiSeq

*RP1, RP2, RPGR, RHO, PRPH2, RP9, IMPDH1, PRPF31, CRB1, PRPF8, TULP1, CA4, PRPF3, ABCA4, RPE65, OFD1, EYS, CERKL, NRL, FAM161A, FSCN2, TOPORS, PRCD, NR2E3, MERTK, USH2A, SNRNP200, SEMA4A, PDE6B, PROM1, KLHL7, PDE6A, RGR, CNGB1, IDH3B, SAG, GUCA1B, CNGA1, BEST1, TTC8, RDH12, C2orf71, ARL6, IMPG2, PDE6G, ZNF513, DHDDS, PRPF6, CLRN1, MAK, C8orf37, CDHR1, RBP3, ROM1, ARL2BP, RLBP1, SPATA7, AIPL1, LRAT, FLVCR1, CYP4V2, KCNJ10, ABHD12, GUCY2D, LCA5, RPGRIP1, CRX, NMNAT1, CEP290, RD3, KCNJ13, IQCB1, GPR143, TYR, OCA2, TYRP1, SLC45A2, SLC24A5, C10orf11, CHN1, CHM, PAX6, TUBA8, HESX1, HPS1, AP3B1, HPS3, HPS4, HPS5, HPS6, DTNBP1, BLOC1S3, BLOC1S6, BBS1, BBS2, BBS4, BBS5, MKKS, BBS7, BBS9, BBS10, TRIM32, BBS12, MKS1, WDPCP, SDCCAG8, LZTFL1, NPHP1, NPHP4, MYO7A, USH1C, CDH23, PCDH15, USH1G, CIB2, GPR98, PDZD7, DFNB31, HARS, SHH, VSX2, ABCB6, STRA6, BCOR, SOX2, OTX2, BMP4, HCCS, VAX1, RARB, HMGB3, MAB21L2, RAX, GDF6, MFRP, PRSS56, GDF3, SMOC1, OPA1, OPA3, TMEM126A, NR2F1, NYX, GRM6, TRPM1, SLC24A1, GPR179, LRIT3, CACNA1F, CABP4, GNAT1, GRK1, CNGB3, CNGA3, GNAT2, PDE6C, PDE6H, OPN1MW, KCNV2, CACNA2D4, PITPNM3, RIMS1, ADAM9, RAX2, GUCA1A, RAB28, TTLL5, POC1B, UNC119, PCYT1A, SC02, INPP5E, TMEM216, AHI1, RPGRIP1L, ARL13B, CC2D2A, TTC21B, KIF7, TCTN1, TMEM237, CEP41, TMEM138, C5orf42, TCTN3, ZNF423, TMEM231, TCTN2,SLC4A11, TGFBI, TACSTD2, KRT12, VSX1, KRT3, DCN, CHST6, PIKFYVE, UBIAD1, FRMD7, KIF21A, TUBB3, PHOX2A, RS1, ELOVL4, FZD4, NDP, LRP5, TSPAN12, VCAN, RDH5, GJA8, CRYGC, CRYBB2, CRYGD, HSF4, EPHA2,CRYAA, CRYBA1, PITX3, BFSP2, GCNT2, GJA3, MIP, CRYAB, CRYBB1, FYCO1, LIM2, CRYGS, MAF, CRYBB3, CRYBA4, VIM, CHMP4B, BFSP1, TDRD7, AGK,CRYGB, NHS, WFS1, CRYBA2, FOXE3, FAM126A, GFER, FTL, JAM3, CTDP1, PTPN11, KRAS, SOS1, RAF1, NRAS, BRAF, MAP2K1, COL2A1, COL11A1, COL11A2, COL9A1, COL9A2, FBN1, CBS, ADAMTS10, LTBP2, FGFR1, FGFR2, FGFR3, PAX3, MITF, SNAI2, EDNRB, EDN3, SOX10, GALC, GJA1, POMT1, TP63, FOXL2, TFAP2A, COL4A1, B3GALTL, OCRL, FREM1, FOXC1, ACTB, ACTG1, PITX2, ABCC2, COL18A1, IFT140, FGF10, HMCN1, FBLN5, CFH, ERCC6, HTRA1, ARMS2, C3, TLR4, CST3, CX3CR1, CFI, C2, CFB, C9, RP1L1, CDH3, POLG, PABPN1, POMT2, POMGNT1, FKTN, FKRP, LARGE, ISPD, POMGNT2, TMEM5, B3GALNT2, POMK, B3GNT1, GMPPB, IGBP1, PIGL, PAX2, SLC4A4, SLC25A4, C10orf2, RRM2B, DNA2, ERCC2, ERCC1, MYOC, OPTN, NTF4, WDR36, CYP1B1, TRIM37, LAMB2, TIMP3, BAP1, TREX1, EFEMP1, ACO2, C1QTNF5, CTC1, RB1, ALMS1, NOD2, CNNM4, TIMM8A, SIL1, ADAMTSL4, RAB3GAP1, RAB3GAP2, RAB18, TBC1D20, CISD2, KIT, ZNF469, PRDM5, ABCC6, GRIP1, FRAS1, FREM2, APTX, SETX, PPT1, TPP1, CLN3, CLN6, DNAJC5, CLN5,MFSD8, CLN8, CTSD, GRN*

Panel 5. sequencing platform: HiSeq

*ABCA4, ABCB6, ABCC6, ABHD12, ACO2, ACOX1, ACTA2, ACTB, ADAM9, ADAMTS18, ADAMTSL4, ADGRV1, AGBL5, AGXT, AHI1, AIPL1, AKT1, ALDH1A3, ALDH3A2, ALG3, ALMS1, AMACR, APOB, APOC2, APOPT1, ARL13B, ARL2BP, ARL6, ATF6, ATOH7, B3GALNT2, B3GLCT, B4GAT1, B9D1, BBIP1, BBS1, BBS10, BBS12, BBS2, BBS4, BBS5, BBS7, BBS9, BCOR, BCS1L, BEST1, BMP4, C12orf57, C1QTNF5, C2orf71, C5orf42, C8orf37, CA4, CABP4, CACNA1F, CACNA2D4, CAPN5, CAV1, CC2D2A, CCDC111, CDH23, CDH3, CDHR1, CEP104, CEP164, CEP290, CEP41, CERKL, CFH, CHD7, CHM, CHST14, CIB2, CLDN19, CLN3, CLN5, CLN6, CLRN1, CNGA1, CNGA3, CNGB1, CNGB3, CNNM4, COA5, COL11A1, COL11A2, COL18A1, COL2A1, COL4A1, COL9A1, COL9A2, COX10, COX14, COX15, COX20, COX6B1, CP, CRB1, CREBBP, CRX, CRYAB, CSPP1, CTC1, CTNNA1, CTNS, CTSD, CYP4V2, DAG1, WHRN, DHDDS, DPM1, DPP6, DRAM2, DTHD1, EDA, EFEMP1, ELOVL4, ERBB3, ERCC2, ERCC3, ERCC5, ERCC6, ERCC8, EXOSC3, EYS, FAM111A, FAM161A, FASTKD2, FBN1, FKRP, FKTN, FLNB, FLVCR1, FLVCR2, FOXRED1, FRMD7, FSCN2, FZD4, G6PC, GDF3, GDF6, GGCX, GMPPB, GNAT1, GNAT2, GNB3, GNPTAB, GPIHBP1, GPR179, GRK1, GRM6, GRN, GSS, GUCA1A, GUCA1B, GUCY2D, HADHA, HADHB, HARS, HBB, HCCS, HGSNAT, HK1, HMX1, HSD11B2, HSD17B10, HSD17B4, IDH3B, IDS, IDUA, IFT122, IFT140, IFT172, IFT27, IFT43, IGFBP7, IMPDH1, IMPG1, IMPG2, INPP5E, INVS, IQCB1, ISPD, ITGA2, ITM2B, JAG1, KCNJ10, KCNJ13, KCNMB1, KCNV2, KIAA0556, KIAA0586, KIF11, KIF7, KIZ, KLHL7, KRIT1, LAMA1, LARGE, LCA5, LPL, LRAT, LRIT3, LRP2, LRP5, LRPAP1, LZTFL1, MAF, MAK, MAN2B1, MCOLN1, MERTK, MFRP, MFSD8, MKKS, MKS1, MMACHC, MTTP, MVK, MYO7A, NAA10, NDP, NDUFA10, NDUFA12, NDUFA2, NDUFA9, NDUFAF2, NDUFAF6, NDUFS3, NDUFS4, NDUFS7, NDUFS8, NEK2, NF2, NMNAT1, NOTCH2, NOTCH3, NPHP1, NPHP3, NPHP4, NR2E3, NRL, NYX, OAT, OCA2, OFD1, OPA3, OPN1LW, OPN1SW, OTX2, P3H2, PANK2, PAX2, PAX6, PCDH15, PCYT1A, PDE6A, PDE6B, PDE6C, PDE6D, PDE6G, PDE6H, PDZD7, PET100, PEX1, PEX10, PEX12, PEX13, PEX14, PEX16, PEX19, PEX2, PEX26, PEX3, PEX5, PEX6, PEX7, PGK1, PHYH, PIEZO2, PIGL, PIK3CA, PITPNM3, PLA2G5, PLK4, PLOD1, PMM2, PNPLA6, POC1B, POGZ, POMGNT1, POMGNT2, POMK, POMT1, POMT2, PORCN, PPT1, PRCD, PROM1, PRPF3, PRPF31, PRPF4, PRPF6, PRPF8, PRPH2, PRSS56, PTEN, RAB28, RAI1, RAX, RAX2, RB1, RBP3, RBP4, RD3, RDH11, RDH12, RDH5, RGR, RGS9, RGS9BP, RHO, RIMS1, RLBP1, RNF113A, RNF216, ROM1, RP1, RP1L1, RP2, RP9, RPE65, RPGR, RPGRIP1, RPGRIP1L, RS1, RTN4IP1, SACS, SAG, SALL1, SALL2, SALL4, SCO2, SDCCAG8, SDHA, SDHAF1, SDHD, SEMA3E, SEMA4A, SHH, SIX6, SLC19A2, SLC24A1, SLC25A15, SLC37A4, SLC38A8, SLC39A5, SLC45A2, SLC7A14, SNRNP200, SPATA7, SPG11, STRA6, SUMF1, SURF1, TACO1, TBX1, TCTN1, TCTN2, TCTN3, TEAD1, TENM3, TFAP2A, TIMM8A, TIMP3, TINF2, TMEM126A, TMEM138, TMEM216, TMEM231, TMEM237, TMEM5, TMEM67, TMEM98, TNFRSF11B, TOPORS, TP53, TPP1, TRAF3IP1, TREX1, TRIM32, TRIM37, TRNT1, TRPM1, TSC1, TSC2, TSPAN12, TTC21B, TTC8, TTLL5, TTPA, TUB, TUBB, TUBGCP4, TUBGCP6, TULP1, UNC119, USH1C, USH1G, USH2A, VCAN, VHL, VPS13B, VSX1, VSX2, WDPCP, WDR19, WFS1, WRN, WWOX, XYLT2, YAP1, ZFYVE26, ZNF408, ZNF423, ZNF513, ZNF644, OPA1, EMC1,CTNNB1*

Panel 6. sequencing platform: HiSeq

*ABCA4, CA4, CTNNA1, GUCY2D, MCOLN1, NF1, PNPLA6, SAG, VCAN, ABCC6, CABP4, CTNNB1, HADHA, MERTK, NF2, POC1B, SANS, VEGF, ABHD12, CACNA1F, CTSA, HARS, MIR204, NMNAT1, POLG, SCO1, WDPCP, ACE, CACNA2D4, CX3CR1, HEXA, MKKS, NOTCH3, POMGNT1, SCO2, WDR19, ACO2, CAPN5, CYP4V2, HEXB, MKS1, NPC1, POMGNT2, SDCCAG8, WHRN, ADAM9, CC2D2A, DAG1, HFE, MMP19, NPC2, POMK, SDHA, XYLT1, ADAMTS18, CCDC28B, DCDC2, HGSNAT, MTATP6, NPHP1, POMT1, SEMA4A, XYLT2, ADGRV1, CCM1, DHDDS, HK1, MTATP8, NPHP3, POMT2, SIX6, ZNF408, AGBL5, CDH23, DHS6S1, HLA-DRB1, MTCO3, NPHP4, PON1, SLC19A2, ZNF423, AGXT, CDH3, DLD, HMCN1, MTFMT, NR2E3, PORCN, SLC24A1, ZNF513, AHI1, CDHR1, DRAM2, HTRA1, MTND1, NRL, PRCD, SLC38A8, AIPL1, CEP104, EFEMP1, IDH3B, MTND2, NYX, PROM1, SLC7A14, ALDH3A2, CEP164, ELOVL4, IFT140, MTND3, OAT, PRPF3, SMPD1, ALMS1, CEP290, EP300, IFT172, MTND4, OFD1, PRPF31, SNRNP200, ANKS6, CEP41, EPO, IFT27, MTND5, OPN1LW, PRPF4, SOD2, APOE, CEP83, ERCC2, IFT43, MTND6, OPN1MW, PRPF6, SPATA7, ARHGEF18, CERKL, ERCC3, IFT74, MTP, OPN1SW, PRPF8, STN1, ARL13B, CFB, ERCC4, IFT81, MTTC, OTX2, PRPH2, SURF1, ARL2BP, CFH, ERCC5, IGFBP7, MTTH, PAX6, PSAP, TBCE, ARL6, CFHR1, ERCC6, IKBKG, MTTK, PCDH15, PTHB1, TCTN2, ARSA, CFHR3, ERCC8, IL1RN, MTTL1, PCYT1A, RAB28, TCTN3, ASAH1, CFI, EYS, IMPDH1, MTTQ, PDCD10, RAX2, TEAD1, ATF6, CHM, FAM111A, IMPG1, MTTS1, PDE6A, RB1, TECT1, ATIC, CIB2, FAM161A, IMPG2, MTTS2, PDE6B, RBP3, TIMP3, ATOH7, CLCN7, FBLN5, INPP5E, MTTV, PDE6C, RBP4, TINF2, B3GALNT2, CLDN19, FKRP, INVS, MTTW, PDE6D, RD3, TLR4, B3GNT1, CLRN1, FKTN, IQCB1, MTTY, PDE6G, RDH11, TMEM138, B9D1, CNGA1, FLVCR1, ISPD, MYO7A, PDE6H, RDH12, TMEM216, BBIP1, CNGA3, FOXRED1, ITM2B, NARS2, PDZD7, RDH5, TMEM231, BBS1, CNGB1, FSCN2, KATNIP, NDP, PEX1, REEP6, TMEM237, BBS10, CNGB3, FZD4, KCNJ13, NDUFA1, PEX10, RGR, TMEM5, BBS12, CNNM4, GALC, KCNV2, NDUFA10, PEX11B, RGS9, TMEM67, BBS2, COL11A1, GDF6, KIAA0586, NDUFA12, PEX12, RGS9BP, TOPORS, BBS4, COL11A2, GLB1, KIF11, NDUFA2, PEX13, RHO, TRAF3IP1, BBS5, COL18A1, GLIS2, KIF7, NDUFA9, PEX14, RIMS1, TRIM32, BBS7, COL2A1, GM2A, KIZ, NDUFAF2, PEX16, RLBP1, TRPM1, BCS1L, COL9A1, GMPPB, KLHL7, NDUFAF5, PEX19, ROM1, TSC1, BEST1, COL9A2, GNAT1, LARGE, NDUFAF6, PEX2, RP1, TSPAN12, BTNL2, COX10, GNAT2, LCA5, NDUFS1, PEX26, RP1L1, TTC21B, C1QTNF5, COX15, GNB3, LOC387715, NDUFS2, PEX3, RP2, TTC8, C2, CP, GNPTAB, LRAT, NDUFS3, PEX5, RP9, TTLL5, C2orf71, CRB1, GPR179, LRIT3, NDUFS4, PEX6, RPE65, TUB, C3, CREBBP, GRHPR, LRP5, NDUFS7, PEX7, RPGR, TUBGCP4, C5orf42, CRX, GRK1, LZTFL1, NDUFS8, PHYH, RPGRIP1, TUBGCP6, C7orf22, CSPP1, GRM6, MAK, NDUFV1, PITPNM3, RPGRIP1L, TULP1, C8orf37, CST3, GUCA1A, MAPKAPK3, NEK2, PLA2G5, RRM2B, USH1C, C9, CTC1, GUCA1B, MAPKBP1, NEU1, PLK4, RS1, USH2A*
