# Supplementary material for: Application of targeted panel sequencing and whole exome sequencing for 76 Chinese families with retinitis pigmentosa
Source: Mol Genet Genomic Med. 2020 Jan 20;8(3):e1131. doi: 10.1002/mgg3.1131 (PMC7057118; doi:10.1002/mgg3.1131)
Supplement: Supplementary file 8 [file MGG3-8-e1131-s008.pdf]

Supplementary Table 3. Genomic information of genes responsible for retinitis pigmentosa in the families in this study

**Disease Category**	**Gene**	**gDNA**	**mRNA**	**Protein**
adRP	*RHO*	NC_000003.11	NM_000539.3	NP_000530.1
	PRPF31	NC_000019.9	NM_015629.3	NP_056444.3
arRP	*USH2A*	NC_000001.10	NM_206933.2	NP_996816.2
	CLRN1	NC_000003.11	NM_174878.2	NP_777367.1
	BBS2	NC_000016.9	NM_031885.3	NP_114091.3
	CYP4V2	NC_000004.11	NM_207352.3	NP_997235.3
	EYS	NC_000006.11	NM_001142800.1	NP_001136272.1
	RPE65	NC_000001.10	NM_000329.2	NP_000320.1
	CNGA1	NC_000004.11	NM_001142564.1	NP_001136036.1
	CNGB1	NC_000016.9	NM_001297.4	NP_001288.3
	PDE6B	NC_000004.12	NM_000283.3	NP_000274.2
	MERTK	NC_000002.11	NM_006343.2	NP_006334.2
	RP1	NC_000008.10	NM_006269.1	NP_006260.1
	CERKL	NC_000002.11	NM_001030311.2	NP_001025482.1
	CRB1	NC_000001.10	NM_201253.2	NP_957705.1
	SLC7A14	NC_000003.11	NM_020949.2	NP_066000.2
xlRP	*RP2*	NC_000023.10	NM_006915.2	NP_008846.2
	RPGR	NC_000023.10	NM_001034853.1	NP_001030025.1

Note: adRP=autosomal dominate retinitis pigmentosa; arRP=autosomal recessive retinitis pigmentosa; and xlRP=X-linked retinitis pigmentosa.
